# Supplementary material for: Efficacy of heparin in respiratory support of near-term rabbits with meconium-induced acute lung injury: Linear regression model analyses
Source: PLoS One. 2026 Mar 24;21(3):e0345718. doi: 10.1371/journal.pone.0345718 (PMC13012467; doi:10.1371/journal.pone.0345718)
Supplement: S1 File — Results of additional analyses. (DOCX) [file pone.0345718.s001.docx]

**S1 Table. Primer sequences (F: forward, R: reverse)**

___________________________________________________________________________

Target genes F/R Sequences (5’-3’)

___________________________________________________________________________

β-actin F ATTGGCATGGCTTTATTCGTG

R GTCACCTTCACCGTTCCAGTTT

SP-A F ATGACTCAAGCGTAATAAATCGTG

R CTCTGGACAAAGCGAGCACA

SP-B F GCTTTACCGCTACTTGCTCACA

R CTTGGACCATCTTCTGTTGCTTT

SP-C F TGGTCCTCGTGGTCGTGGTG

R GCTGGTAGTCGCAGGTGACAATG

SP-D F CCTGAGCATGACTGACACCAAGAC

R CACAAGGCGTTCCTCTCCACAAG

IGF-1 F CATCCTGTCCTCCTCGCATC

R ACTGAAGAGCATCCACCAGC

IGF-2 F TCTCTGACGACCGTGCTTCCG

R GGCTGGTGATGCTTGGCTTCC

VEGF F ACGCCTACCCACATACATAACG

R CTCCCAGCTCCAGTCCACAG

KGF F TTCCAGCCCTGAGCGACATA

R TCCAACTGCCACAGTCCTGA

eNOS F GTGGTAACCAGCACCTTTGG

R GCAGGAAACACTATTGAAGC

iNOS F GCGCCTGCATGACATTGAGA

R TGAGGACGCAGCCAAACACT

AQP-5 F GCCGTGGTGGTGGAGTTGATC

R CATGGAGCAGCCGGTGAAGTAG

Na^+^-K^+^ ATPase α1 F TCTTCCTCATTGGCATCATCGTAGC

R GTCTCCACGGCTTCCAAGTTCTTC

syndecan-1 F CTTTGCCGTGTGCCTGGTG

R GCGTAGAACTCCTCTTGCTTGG

TF F AACAGCCACGACAAACACTAATGAG

R CCGAGAGGGAATCACTGCTTGG

Ang-1 F CCTGCCTCGCTGCCATTGTG

R CAGTTGCCGTCCTGTTCTGGAAG

Ang-2 F CCAGTGGCATCTACACGCTAACG

R CCATCTTCCCGCCGCTGAATG

Tie-1 F AGAGGAAGCCAGTGCCAGGAAG

R ACCGATCACAAGTGCCACCATTC

Tie-2 F CACTCCCAGAATCTCAAGCACCAG

R GCACTGTCAGGCAGGTCATTCC

TLR-2 F GGTGTCTGGCGTGTGCTGTG

R AGTAGGAGTCGTGCTCGCTGTAG

TLR-4 F GTATCGCCTTCTCAGCAGGAACAC

R TGAGCCGTCTCCAGAAGATATGCC

NF-κB F GATGTGAAGATGCTCCTGGCTGTC

R CGGTGGATGATTGCTAGGTGTAAGAC

MyD88 F CTGAAGCTGTGCGTGTCTGACC

R TCCGAGACGACCACCACCATC

TNF-α F GGCATGAAGCTCACGGACAACC

R GCCTTGACCGCTGAAGAGAACC

IL-1β F CAGGCTCCAGGATGCACAACAG

R CACGCAGGACAGGTACAGATTCTTC

IL-6 F GAAGAAGCCACCCTCAAGCC

R CCATGAAATTCCGCAAGCAA

IL-8 F TTGGTCAGGCCATGAGTTCC

R TGTGCCCTCACAACATCCCT

___________________________________________________________________________

Abbreviations: SP, surfactant protein; IGF, insulin-like growth factor; VEGF, vascular endothelial GF; KGF, keratinocyte GF; eNOS, endothelial nitric oxide synthase; iNOS, inducible nitric oxide synthase; AQP-5, aquaporin-5; TF, tissue factor; Ang, angiopoietin; TLR, toll-like receptor; MyD88, myeloid differentiation primary response protein 88; NF-κB, nuclear transcript factor kappa B; TNF-α, tumor necrosis factor α; IL, interleukin.

**S2 Table. Blood gas analysis among different groups.**

___________________________________________________________________________

Group n pH PCO_2_  LAC

___________________________________________________________________________

A. Treatment groups

C0 15 7.17 + 0.11 72.7 + 14.4 7.83 + 5.15

C 13 7.32 + 0.13^§^  61.3 + 16.5 4.42 + 1.39

M 10 7.13 + 0.23 83.7 + 33.3 6.47 + 5.32

MS 15 7.32 + 0.09^§§^ 62.1 + 14.9 3.99 + 1.41

MH 17 7.26 + 0.10 72.8 + 25.8 4.19 + 2.17

MSH 18 7.27 + 0.12 66.9 + 17.1 3.67 + 1.57

MN 16 7.35 + 0.08^§^^§§^ 58.2 + 13.6^§^^§^  4.20 + 1.31

MSN 15 7.39 + 0.06^§§§^ 53.4 + 11.0^§§^ ^§^ 4.08 + 0.97

MHN 17 7.31 + 0.11^§§^ 66.6 + 18.5 4.26 + 2.38

MSHN 18 7.37 + 0.06^§§§^ 55.0 + 6.93^§§§^ 4.31 + 1.52

___________________________________________________________________________

B. Pooled treatment groups

all-PS 66 7.34 + 0.10^*^ 59.5 + 13.9^**^ 4.01 + 1.39

non-PS 60 7.28 + 0.15 69.0 + 23.7 4.59 + 2.88

all-UFH 70 7.30 + 0.11 65.2 + 19.0 4.10 + 1.91

non-UFH 56 7.31 + 0.15 62.5 + 20.7 4.52 + 2.59

all-iNO 66 7.35 + 0.08^***^  58.4 + 13.9^**^ ^*^ 4.22 + 1.62

non-iNO 60 7.26 + 0.14 70.2 + 23.2 4.36 + 2.78

___________________________________________________________________________

Values are mean + SD, n = 10-18. For group definitions in A and B, see Table 1 legends. ^§^*p* < 0.05, ^§§^*p* < 0.01, ^§§§^*p* < 0.001 vs. M. ^*^*p* < 0.05, ^**^*p* < 0.01, ^***^*p* < 0.001 vs. corresponding pooled non-designated drug group.

**S3 Table. Changes of Cdyn and** **survival rate of all the groups during ventilation time**

____________________________________________________________________________________________________________________

Cdyn (ml/kg/cmH_2_O)

survival rate (%)

_____________________________________________________________________________________________________________

Group C M MS MH MSH MN MSN MHN MSHN

N 21 21 20 20 20 20 20 21 21

Time

___________________________________________________________________________________________________________________

0.25 h 0.52 + 0.22 0.36 + 0.19 0.42 + 0.19 0.39 + 0.18 0.30 + 0.11^^^^ 0.36 + 0.12 0.40 + 0.16 0.40 + 0.11 0.41 + 0.18

(100%) (100%) (100%) (100%) (100%) (100%) (100%) (100%) (100%)

0.5 h  0.62 + 0.19 0.47 + 0.26 0.60 + 0.22 0.49 + 0.25 0.45 + 0.18 0.51 + 0.15 0.58 + 0.24 0.51 + 0.22 0.55 + 0.20

(100%) (100%) (100%) (100%) (100%) (100%) (100%) (100%) (100%)

0.75 h  0.69 + 0.23 0.53 + 0.26 0.60 + 0.25 0.54 + 0.35 0.56 + 0.20 0.56 + 0.19 0.63 + 0.24 0.54 + 0.21 0.60 + 0.22

(100%) (100%) (100%) (100%) (100%) (100%) (100%) (100%) (100%)

1 h  0.65 + 0.17 0.53 + 0.20 0.72 + 0.26 0.61 + 0.32 0.69 + 0.23 0.54 + 0.26 0.65 + 0.23 0.62 + 0.23 0.54 + 0.14

(100%) (100%) (100%) (100%) (100%) (100%) (100%) (100%) (100%)

1.5 h  0.66 + 0.15 0.52 + 0.20 0.68 + 0.22 0.57 + 0.30 0.77 + 0.24^§§^ 0.53 + 0.22 0.65 + 0.21 0.61 + 0.21 0.61 + 0.18

(100%) (100%) (100%) (100%) (100%) (100%) (100%) (100%) (100%)

2 h 0.76 + 0.21 0.53 + 0.21^^^ 0.57 + 0.23 0.58 + 0.25 0.67 + 0.22 0.60 + 0.26 0.68 + 0.23 0.65 + 0.23 0.67 + 0.30

(100%) (100%) (100%) (100%) (100%) (100%) (100%) (100%) (100%)

3 h  0.75 + 0.24 0.46 + 0.18^^^^ 0.49 + 0.22^^^^ 0.62 + 0.25 0.56 + 0.26  0.56 + 0.27 0.56 + 0.23 0.59 + 0.26 0.49 + 0.16^^^^

(100%) (86%) (100%) (100%) (100%) (100%) (100%) (100%) (100%)

4 h  0.68 + 0.22^§§^ 0.43 + 0.16 0.65 + 0.21^§^ 0.76 + 0.20^§§§^0.66 + 0.24^§^  0.78 + 0.52^§§^ 0.68 + 0.22 0.79 + 0.24^§§^ 0.65 + 0.20

(100%) (81%) (100%) (100%) (100%) (100%) (100%) (100%) (100%)

5 h 0.68 + 0.19 0.52 + 0.22 0.66 + 0.18 0.70 + 0.18 0.69 + 0.27 0.75 + 0.39 0.66 + 0.21 0.73 + 0.24 0.66 + 0.23

(100%) (71%) (100%) (100%) (100%) (95%) (100%) (100%) (100%)

6 h  0.71 + 0.19 0.53 + 0.19 0.75 + 0.24^§^ 0.70 + 0.19 0.66 + 0.26 0.71 + 0.38 0.67 + 0.16 0.67 + 0.19 0.69 + 0.19

(100%) (67%) (100%) (95%) (100%) (95%) (100%) (100%) (100%)

7 h 0.67 + 0.17 0.60 + 0.18 0.69 + 0.24 0.78 + 0.19 0.65 + 0.25 0.72 + 0.38 0.71 + 0.24 0.69 + 0.22 0.66 + 0.29

(100%) (67%) (95%) (95%) (100%) (95%) (100%) (100%) (100%)

8 h 0.73 + 0.18 0.59 + 0.20 0.80 + 0.28 0.77 + 0.19 0.74 + 0.25 0.69 + 0.37 0.73 + 0.23 0.81 + 0.27 0.72 + 0.31

(100%) (67%) (90%) (95%) (95%) (95%) (100%) (95%) (100%)

9 h 0.76 + 0.26 0.59 + 0.17 0.83 + 0.29 0.73 + 0.16 0.73 + 0.30 0.77 + 0.35 0.81 + 0.23 0.86 + 0.26 0.79 + 0.32

(100%) (67%) (85%) (95%) (95%) (95%) (90%) (95%) (100%)

10 h 0.78 + 0.29 0.56 + 0.24 0.89 + 0.24^§^ 0.81 + 0.28 0.78 + 0.25 0.86 + 0.37 0.86 + 0.27 0.89 + 0.27^§^ 0.81 + 0.25

(100%)^§^^§^ (67%) (85%) (95%)^§^ (95%)^§^ (95%)^§^ (85%) (95%)^§^ (100%)^§§^

_________________________________________________________________________________________________________________

Values are mean + SD, n = 20-21. For group definitions in A and B, see Table 1 legends. ^^^*p* < 0.05, ^^^^*p* < 0.01 vs. C; ^§^*p* < 0.05, ^§§^*p* < 0.01, ^§§§^*p* < 0.001 vs. M.

**S4 Table. Morphometric measurements of alveolar expansion**

___________________________________________________________________________

Group n Vv CV (Vv)

___________________________________________________________________________

A. Treatment groups

C0 7  0.53 + 0.07 0.19 + 0.03

C 8 0.48 + 0.04 0.24 + 0.03

M 8 0.49 + 0.03 0.29 + 0.04

MS 8 0.59 + 0.04^^^^^§§^ 0.23 + 0.04

MH 8 0.58 + 0.06^^^§^ 0.24 + 0.04

MSH 8 0.59 + 0.07^^^§^ 0.23 + 0.06

MN 8 0.61 + 0.05^^^^§§§※&^ 0.20 + 0.05^§§ §^

MSN 8 0.68 + 0.04^^^^§§§^ 0.17 + 0.02^^^§§§^

MHN 8 0.63 + 0.05^^^^§§§^ 0.20 + 0.05^§§§^

MSHN 8 0.68 + 0.04^^^^§§§^ 0.16 + 0.02^^^^^^§§§^

___________________________________________________________________________

B. Pooled treatment groups

all-PS 32 0.63 + 0.07^**^  0.20 + 0.05^*^

non-PS 32 0.58 + 0.07 0.23 + 0.06

all-UFH 32 0.62 + 0.07 0.20 + 0.06

non-UFH 32 0.59 + 0.08 0.22 + 0.06

all-iNO 32 0.65 + 0.05^***^ 0.18 + 0.04^***^

non-iNO 32 0.56 + 0.07 0.25 + 0.05

___________________________________________________________________________

Values are mean + SD, n = 7-8. For group definitions in A and B, see Table 1 legends. Vv, volume density of alveolar expansion; CV (Vv), variation of alveolar aeration; LIS_total_, total lung injury score. ^^^^*p* < 0.01, ^^^^^*p* < 0.001 vs. C; ^§^*p* < 0.05, ^§§^*p* < 0.01, ^§§§^*p* < 0.001 vs. M; ^※^*p* < 0.05 vs. MSN; ^&^*p* < 0.05 vs. MSHN. ^*^*p* < 0.05, ^**^*p* < 0.01, ^***^*p* < 0.001 vs. corresponding pooled non-designated drug group.

**S5 Table. Lung injury score categorized by injury pattern in items.**

___________________________________________________________________________

Bronchiolar

epithelial Meconium

Group n Edema Hemorrhage Inflammation disruption distribution

___________________________________________________________________________

A. Treatment groups

C0 7 0.0 (0-1) 0.0 (0-1) 0.0 (0-0) 0.0 (0-1) 0.0 (0-0)

C 8 0.0 (0-1) 1.0 (0-1) 0.0 (0-1) ^§^ 1.0 (0-1) 0.0 (0-0)^§§§^

M 8  1.0 (0-4) 0.5 (0-1) 1.5 (1-3) 1.0 (0-2) 3.5 (2-4)

MS 8 0.0 (0-1) 0.0 (0-1) 1.0 (0-2) 1.0 (0-1) 3.0 (2-4) ^^^

MH 8 0.0 (0-1) 0.5 (0-1) 1.0 (0-2) 1.0 (0-2) 3.0 (3-4)^^^^

MSH 8 0.0 (0-1) 0.5 (0-1) 1.0 (0-1) 1.0 (0-1) 3.0 (2-4)^^^^

MN 8 0.0 (0-1) 0.0 (0-1) 0.0 (0-0) ^§§§^ 0.5 (0-1) 3.0 (2-3) ^^^

MSN 8  0.0 (0-1) 0.0 (0-1) 0.0 (0-0)^§§§^ 0.0 (0-1) 3.0 (1-4)^^^^

MHN 8 0.0 (0-1) 0.5 (0-1) 0.0 (0-1) ^§§§^ 0.0 (0-1) 3.0 (2-4)^^^

MSHN 8 0.0 (0-1) 0.0 (0-1) 0.0 (0-0)^§§§^ 0.0 (0-1) 3.0 (1-4)^^^^

___________________________________________________________________________

B. Pooled treatment groups

all-PS 32 0.0 (0-1) 0.0 (0-1) 0.0 (0-2) 1.0 (0-1) 3.0 (1-4)

non-PS 32 0.0 (0-4) 0.0 (0-1) 0.0 (0-3) 1.0 (0-2) 3.0 (2-4)

all-UFH 32 0.0 (0-1) 0.0 (0-1) 0.0 (0-2) 0.5 (0-2) 3.0 (1-4)

non-UFH 32 0.0 (0-4) 0.0 (0-1) 0.0 (0-3) 1.0 (0-2) 3.0 (1-4)

all-iNO 32 0.0 (0-1) 0.0 (0-1) 0.0 (0-1) ^***^ 0.0 (0-1) ^**^ 3.0 (1-4)

non-iNO 32 0.0 (0-4) 0.0 (0-1) 1.0 (0-3) 1.0 (0-2) 3.0 (2-4)

___________________________________________________________________________

Values are median (range, minimum - maximum). n = 7-8. For group definitions in A and B, see Table 1 legends. ^§^*p* < 0.05, ^§§§^*p* < 0.001 vs. M; ^^^*p* < 0.05, ^^^^*p* < 0.01 vs. C. ^**^*p* < 0.01, ^***^*p* < 0.001 vs. corresponding pooled non-designated drug group.

**S6 Table.** **Biochemical analysis of lung homogenates.**

A. LH

___________________________________________________________________________

Group n TPL DSPC DSPC/TPL

mg/kg mg/kg %

___________________________________________________________________________

a. Treatment groups

C0 12 411.0 + 123.6 104.8 + 46.3 25.6 + 7.3

C 10 436.2 + 99.0 114.5 + 44.5 25.7 + 5.1

M 12 439.9 + 110.4 124.2 + 36.7 28.8 + 8.2

MS 11 488.8 + 119.2 158.7 + 73.3 32.5 + 12.0^£^

MH 10 469.7 + 160.0 96.6 + 43.7 20.3 + 4.8

MSH 11 487.3 + 161.2 161.2 + 63.7 33.6 + 10.6^£^

MN 12 488.0 + 112.9 113.5 + 31.2 24.1 + 7.7

MSN 11 495.1 + 118.8 161.2 + 58.5 34.0 + 13.2

MHN 12 461.7 + 133.8 111.0 + 40.1 24.0 + 4.8

MSHN 13 507.1 + 163.6 151.5 + 59.5 30.8 + 9.1

___________________________________________________________________________

b. Pooled treatment groups

all-PS 46 495.1 + 138.8 157.9 + 61.8^***^ 32.6 + 10.9^***^

non-PS 46 464.6 + 126.1 112.0 + 37.9 24.5 + 7.1

all-UFH 46 482.4 + 150.8 131.3 + 57.7 27.4 + 9.2

non-UFH 46 477.3 + 113.6 138.5 + 54.5 29.7 + 10.8

all-iNO 48 488.2 + 131.5 134.1 + 52.3 28.1 + 9.8

non-iNO 44 470.7 + 135.1 135.8 + 60.3 29.0 + 10.4

___________________________________________________________________________

B. LH+BALF

_____________________________________________________________________

Group n TPL DSPC DSPC/TPL

mg/kg mg/kg %

_____________________________________________________________________

c. Treatment groups

C0 12 414.5 + 124.2 106.4 + 46.2 25.8 + 7.3

C 10 439.6 + 98.0 115.9 + 44.2 25.8 + 5.1

M 12 441.2 + 110.0 124.8 + 36.7 28.8 + 8.2

MS 11 492.2 + 119.5 160.1 + 73.3 32.6 + 12.0^£^

MH 10 471.7 + 160.1 97.3 + 43.9 20.4 + 4.8

MSH 11 489.7 + 160.9 162.3 + 63.5 33.6 + 10.5^£^

MN 12 489.5 + 112.8 114.3 + 31.3 24.2 + 7.8

MSN 11 497.4 + 118.7 162.4 + 58.4 34.0 + 13.2

MHN 12 463.3 + 133.7 111.7 + 40.1 24.0 + 4.8

MSHN 13 509.8 + 163.8 152.8 + 59.4 30.9 + 9.0

_____________________________________________________________________

d. Pooled treatment groups

all-PS 46 497.8 + 138.8 159.1 + 61.7^***^ 32.7 + 10.9^***^

non-PS 46 466.2 + 126.0 112.7 + 37.9 24.5 + 7.1

all-UFH 46 484.6 + 150.8 132.3 + 57.8 27.5 + 9.1

non-UFH 46 479.4 + 113.6 139.5 + 54.6 29.7 + 10.8

all-iNO 48 490.3 + 131.6 135.1 + 52.4 28.2 + 9.8

non-iNO 44 473.0 + 135.1 136.7 + 60.4 29.0 + 10.3

_____________________________________________________________________

Values are mean + SD, n = 10-13. For group definitions in A and B, see Table 1 legends. ^£^*p* < 0.05 vs MH. ^***^*p* < 0.001 vs. corresponding pooled non-designated drug group.

**S7 Table. Relative rate of change for each group in the total lung phospholipid pools** ___________________________________________________________________________

TPL DSPC (DSPC/TPL) OR (95%CI)

/TPL /DSPC /(DSPC/TPL)

___________________________________________________________________________

A. UFH

MH/M 1.07 0.78 0.71 0.670 (0.667-0.673)

MSH/MS 0.99 1.01 1.03 1.028 (1.025-1.032)

MHN/MN 0.95 0.98 0.99 1.043 (1.039-1.047)

MSHN/MSN 1.02 0.94 0.91 0.883 (0.880-0.886)

___________________________________________________________________________

B. PS

MS/M 1.12 1.28 1.13 1.222 (1.218 -1.227)

MSH/MH 1.04 1.67 1.65 1.907 (1.900 -1.915)

MSN/MN 1.02 1.42 1.40 1.591 (1.585 -1.597)

MSHN/MHN 1.10 1.37 1.29 1.347 (1.342 -1.352)

___________________________________________________________________________

C. iNO

MN/M 1.11 0.92 0.84 0.786 (0.782-0.789)

MSN/MS 1.01 1.01 1.04 1.006 (1.002-1.009)

MHN/MH 0.98 1.15 1.18 1.222 (1.217-1.228)

MSHN/MSH 1.04 0.94 0.92 0.863 (0.860-0.866)

___________________________________________________________________________

For group definitions in A, B and C, see Table 1 legends. Estimation using the test of proportions to find the 95% confidence limits (interval, 95%CI) for the ratio between group means.

e.g. MS/M for Odds ratio: ${\left[ \frac{[DSPC]}{([TPL]-[DSPC])} \right]_{\mathrm{MS}}}/{\left[ \frac{[DSPC]}{([TPL]-[DSPC])} \right]_{M}}$

SE ($\log_{e} OR$) =$\sqrt{\frac{1}{n_{1}P_{1}(1-P_{1})}+\frac{1}{{n_{2}P}_{2}(1-P_{2})}}$ or

SE ($\log_{e} OR$) =$\sqrt{\left[ \frac{1}{n\times[DSPC]([TPL]-[DSPC])} \right]_{\mathrm{MS}}+ \left[ \frac{1}{n\times[DSPC]([TPL]-[DSPC])} \right]_{M}}$

The 95% confidence interval (95%CI) for the $\log_{e} OR$is given by: $\log_{e} OR-$1.96 SE ($\log_{e} OR$) to $\log_{e} OR+$1.96 SE ($\log_{e} OR$).

The 95%CI for the OR is derived by taking the exponential of the result of 95%CI for the $\log_{e} OR$

Assume: *P*_1_ = [DSPC]_MS_; 1 - *P*_1_ = ([TPL]-[DSPC])_MS_; *P*_2_ = [DSPC]_M_, 1 - *P*_2_ = ([TPL] -[DSPC])_M_; n_1_ = 11 (MS), n_2_ = 12 (M) [see S6. B Table (BALF + LH) c (Treatment groups)].

[TPL] and [DSPC] denote the amount of TPL and DSPC corrected by birth weight in kg, mg/kg (see S6. B Table); M and MS denote group M and MS (see Table 1 legends); The n is the number of animals per group analyzed for the phospholipid amount.

**S8 Table. The mRNA expression of molecules from lung tissue.**

A. Proinflammatory cytokines and mediators

___________________________________________________________________________

Group n TLR-2 TLR-4 MyD88 NF-κB

___________________________________________________________________________

a. Treatment groups

C0 8 1.00 + 0.00 1.00 + 0.00 1.00 + 0.00 1.00 + 0.00

C 9 1.45 + 1.11 1.17 + 0.60 1.23 + 0.48 0.95 + 0.42

M 13 1.23 + 0.96 1.20 + 0.73 1.28 + 0.62 1.08 + 0.57

MS 10 0.97 + 0.69 1.03 + 0.44 1.38 + 0.53 1.04 + 0.41

MH 12 1.03 + 1.17 0.99 + 0.52 1.20 + 0.52 0.95 + 0.46

MSH 11 1.07 + 0.68 1.01 + 0.28 1.16 + 0.41 0.99 + 0.39

MN 11 0.70 + 0.59 0.87 + 0.31 0.77 + 0.16 0.69 + 0.13

MSN 11 0.52 + 0.37 0.91 + 0.36 0.80 + 0.26 0.73 + 0.28

MHN 13 0.79 + 0.74 0.95 + 0.57 0.86 + 0.54 0.76 + 0.54

MSHN 13 0.86 + 0.88 1.00 + 0.47 0.88 + 0.39 0.86 + 0.48

___________________________________________________________________________

b. Pooled treatment groups

all-PS 45 0.85 + 0.70 0.99 + 0.38 1.04 + 0.45 0.90 + 0.40

non-PS 49 0.95 + 0.89 1.01 + 0.56 1.03 + 0.53 0.87 + 0.48

all-UFH 49 0.93 + 0.87 0.99 + 0.46 1.02 + 0.48 0.88 + 0.47

non-UFH 45 0.87 + 0.73 1.01 + 0.50 1.06 + 0.51 0.89 + 0.42

all-iNO 48 0.73 + 0.68^*^ 0.94 + 0.43 0.83 + 0.37^***^ 0.76 + 0.40^**^

non-iNO 46 1.08 + 0.89 1.06 + 0.52 1.25 + 0.52 1.01 + 0.46

___________________________________________________________________________

___________________________________________________________________________

Group n TNF-α IL-1β IL-6 IL-8

___________________________________________________________________________

a. Treatment groups

C0 8 1.00 + 0.00 1.00 + 0.00 1.00 + 0.00 1.00 + 0.00

C 9 1.18 + 1.07 2.88 + 1.26 1.16 + 0.96 0.89 + 0.47

M 13 1.01 + 0.84 4.31 + 4.46 1.09 + 1.01 0.97 + 0.52

MS 10 0.71 + 0.62 3.51 + 1.60 0.82 + 0.86 0.79 + 0.42

MH 12 0.87 + 1.04 3.53 + 2.13 0.97 + 1.31 0.82 + 0.60

MSH 11 0.93 + 0.63 3.84 + 1.91 1.00 + 0.72 1.06 + 0.39

MN 11 0.48 + 0.27 1.97 + 1.26 0.54 + 0.32 0.53 + 0.26

MSN 11 0.44 + 0.27 1.64 + 1.01 0.47 + 0.31 0.48 + 0.18

MHN 13 0.61 + 0.68 2.24 + 2.16 0.72 + 0.77 0.61 + 0.48

MSHN 13 0.73 + 0.84 2.37 + 1.92 0.81 + 0.75 0.68 + 0.50

___________________________________________________________________________

b. Pooled treatment groups

all-PS 45 0.70 + 0.64 2.80 + 1.83 0.78 + 0.69 0.75 + 0.44

non-PS 49 0.75 + 0.77 3.04 + 2.91 0.84 + 0.93 0.74 + 0.50

all-UFH 49 0.78 + 0.80 2.95 + 2.09 0.87 + 0.90 0.78 + 0.51

non-UFH 45 0.67 + 0.60 2.91 + 2.80 0.75 + 0.73 0.70 + 0.42

all-iNO 48 0.57 + 0.58^*^ 2.08 + 1.66^***^ 0.65 + 0.60^*^ 0.58 +0.39^***^

non-iNO 46 0.89 + 0.79 3.82 + 2.80 0.98 + 0.98 0.91 + 0.49

___________________________________________________________________________

B. Endothelial cell proliferation, injury, and coagulation-related factors

___________________________________________________________________________

Group n Ang-1 Ang-2 Tie-1 Tie-2

___________________________________________________________________________

a. Treatment groups

C0 6 1.00 + 0.00 1.00 + 0.00 1.00 + 0.00 1.00 + 0.00

C 8 0.97 + 0.19 1.56 + 0.38 0.44 + 0.13 0.94 + 0.31

M 8 0.90 + 0.74 1.28 + 0.82 0.80 + 0.99 0.95 + 0.97

MS 9 1.34 + 0.50 2.20 + 1.17 0.69 + 0.33 1.58 + 0.55

MH 8 1.02 + 0.56 1.93 + 1.59 0.44 + 0.22 1.02 + 0.42

MSH 10 1.08 + 0.34 1.69 + 1.07 0.51 + 0.30 1.09 + 0.37

MN 10 1.18 + 0.39 1.85 + 0.70 0.50 + 0.20 0.97 + 0.25

MSN 8 1.42 + 1.04 2.49 + 0.96 0.51 + 0.32 0.97 + 0.65

MHN 6 1.11 + 0.47 1.45 + 0.37 0.39 + 0.15 0.79 + 0.28

MSHN 8 1.45 + 0.78 2.25 + 0.93 0.62 + 0.34 1.11 + 0.69

___________________________________________________________________________

b. Pooled treatment groups

all-PS 35 1.31 + 0.68 2.13 + 1.04 0.58 + 0.31 1.19 + 0.59

non-PS 32 1.06 + 0.53 1.66 + 0.98 0.54 + 0.52 0.94 + 0.54

all-UFH 32 1.16 + 0.55 1.85 + 1.10 0.50 + 0.27 1.02 + 0.46

non-UFH 35 1.21 + 0.68 1.96 + 0.99 0.62 + 0.52 1.12 + 0.67

all-iNO 32 1.29 + 0.69 2.04 + 0.84 0.51 + 0.27 0.97 + 0.50

non-iNO 35 1.09 + 0.54 1.79 + 1.18 0.61 + 0.53 1.17 + 0.63

___________________________________________________________________________

________________________________________________

Group n syndecan-1 TF

________________________________________________

a. Treatment groups

C0 8 1.07 + 0.62 1.00 + 0.00

C 9 0.98 + 0.56 0.79 + 0.36

M 13 0.96 + 0.56 1.15 + 0.39

MS 10 1.12 + 0.73 0.96 + 0.58

MH 12 1.04 + 0.74 0.89 + 0.50

MSH 11 1.06 + 0.66 0.86 + 0.37

MN 11 1.13 + 0.54 1.05 + 0.38

MSN 11 0.94 + 0.60 1.20 + 0.36

MHN 13 0.90 + 0.39 0.83 + 0.33

MSHN 13 1.15 + 0.57 1.03 + 0.51

_______________________________________________

b. Pooled treatment groups

all-PS 45 1.07 + 0.62 1.02 + 0.46

non-PS 49 1.00 + 0.56 0.98 + 0.41

all-UFH 49 1.04 + 0.59 0.90 + 0.43^*^

non-UFH 45 1.03 + 0.59 1.09 + 0.43

all-iNO 48 1.03 + 0.52 1.02 + 0.41

non-iNO 46 1.04 + 0.65 0.97 + 0.46

________________________________________________

C. Other molecules

___________________________________________________________________________

Group n SP-A SP-B SP-C SP-D

___________________________________________________________________________

a. Treatment groups

C0 8 1.00 + 0.00 1.00 + 0.00 1.00 + 0.00 1.00 + 0.00

C 9 1.09 + 0.55 1.30 + 0.55 0.76 + 0.40 0.78 + 0.51

M 13 1.43 + 1.30 1.36 + 1.03 1.49 + 1.58 1.11 + 0.83

MS 10 1.26 + 0.66 1.10 + 0.57 1.30 + 0.87 0.96 + 0.79

MH 12 1.16 + 0.79 1.18 + 0.90 1.13 + 0.80 0.85 + 0.70

MSH 11 1.18 + 1.06 1.04 + 0.44 0.93 + 0.59 0.80 + 0.47

MN 11 1.33 + 1.32 1.28 + 1.38 1.48 + 1.02 0.96 + 0.81

MSN 11 0.92 + 0.51 0.76 + 0.52 1.02 + 0.67 0.70 + 0.41

MHN 13 0.88 + 0.67 0.81 + 0.48 1.21 + 0.97 0.80 + 0.75

MSHN 13 1.23 + 0.86 0.89 + 0.67 1.41 + 0.95 1.02 + 0.89

___________________________________________________________________________

b. Pooled treatment groups

all-PS 45 1.15 + 0.79 0.94 + 0.56 1.17 + 0.79 0.87 + 0.67

non-PS 49 1.20 + 1.04 1.15 + 0.98 1.32 + 1.12 0.93 + 0.76

all-UFH 49 1.11 + 0.83 0.97 + 0.65 1.18 + 0.84 0.87 + 0.71

non-UFH 45 1.24 + 1.02 1.14 + 0.95 1.33 + 1.10 0.94 + 0.73

all-iNO 48 1.09 + 0.88 0.93 + 0.82 1.28 + 0.91 0.87 + 0.73

non-iNO 46 1.26 + 0.98 1.18 + 0.78 1.22 + 1.04 0.93 + 0.70

___________________________________________________________________________

___________________________________________________________________________

Group n IGF-1 IGF-2 VEGF KGF

___________________________________________________________________________

a. Treatment groups

C0 8 1.00 + 0.00 1.00 + 0.00 1.00 + 0.00 1.00 + 0.00

C 9 0.74 + 0.34 1.28 + 0.47 1.12 + 0.57 0.56 + 0.58

M 13 0.81 + 0.38 1.20 + 0.58 1.13 + 0.62 0.46 + 0.40

MS 10 0.84 + 0.30 1.15 + 0.37 1.13 + 0.50 0.58 + 0.29

MH 12 0.82 + 0.70 1.30 + 0.68 1.29 + 0.94 0.67 + 0.63

MSH 11 0.60 + 0.27 1.20 + 0.55 1.24 + 0.59 0.47 + 0.30

MN 11 0.98 + 0.35 0.90 + 0.30 0.76 + 0.21 0.41 + 0.23

MSN 11 0.90 + 0.52 0.93 + 0.31 0.73 + 0.26 0.32 + 0.20

MHN 13 0.79 + 0.28 0.88 + 0.48 0.89 + 0.82 0.63 + 0.96

MSHN 13 0.75 + 0.31 0.87 + 0.15 0.88 + 0.43 0.48 + 0.33

___________________________________________________________________________

b. Pooled treatment groups

all-PS 45 0.77 + 0.37 1.03 + 0.38 0.99 + 0.49 0.46 + 0.29

non-PS 49 0.85 + 0.45 1.07 + 0.55 1.02 + 0.72 0.55 + 0.62

all-UFH 49 0.74 + 0.42 1.05 + 0.52 1.07 + 0.73 0.56 + 0.61

non-UFH 45 0.88 + 0.39 1.05 + 0.42 0.94 + 0.47 0.44 + 0.30

all-iNO 48 0.85 + 0.37 0.89 + 0.32^***^ 0.82 + 0.50^**^ 0.47 + 0.54

non-iNO 46 0.77 + 0.45 1.21 + 0.55 1.20 + 0.67 0.54 + 0.43

___________________________________________________________________________

___________________________________________________________________________

Group n AQP-5 Na+-K+ ATPase α1 eNOS iNOS

___________________________________________________________________________

a. Treatment groups

C0 8 1.00 + 0.00 1.00 + 0.00 1.00 + 0.00 1.00 + 0.00

C 9 1.03 + 0.67 0.89 + 0.38 0.74 + 0.61 1.09 + 1.64

M 13 1.00 + 0.54 1.06 + 0.36 0.78 + 0.74 1.08 + 1.29

MS 10 1.17 + 0.43 1.07 + 0.46 0.56 + 0.22 0.86 + 1.01

MH 12 1.10 + 0.61 0.91 + 0.41 0.46 + 0.30 0.88 + 1.27

MSH 11 1.21 + 0.86 0.92 + 0.39 0.50 + 0.21 0.73 + 0.78

MN 11 0.93 + 0.22 0.67 + 0.18 0.37 + 0.23 0.71 + 0.38

MSN 11 0.79 + 0.21 0.60 + 0.19^§^ 0.31 + 0.22 0.58 + 0.50

MHN 13 1.01 + 0.85 0.71 + 0.48 0.42 + 0.45 0.80 + 1.02

MSHN 13 1.32 + 0.86 0.77 + 0.41 0.46 + 0.25 1.12 + 1.00

___________________________________________________________________________

b. Pooled treatment groups

all-PS 45 1.13 + 0.68 0.83 + 0.40 0.45 + 0.24 0.84 + 0.85

non-PS 49 1.01 + 0.59 0.84 + 0.40 0.51 + 0.50 0.88 + 1.05

all-UFH 49 1.16 + 0.79 0.82 + 0.42 0.46 + 0.31 0.89 + 1.01

non-UFH 45 0.97 + 0.40 0.85 + 0.38 0.52 + 0.47 0.82 + 0.89

all-iNO 48 1.02 + 0.66 0.69 + 0.35^***^ 0.39 + 0.30^*^ 0.82 + 0.80

non-iNO 46 1.12 + 0.61 0.99 + 0.40 0.58 + 0.45 0.90 + 1.09

___________________________________________________________________________

Values are expressed as means and SD of 2^−ΔΔCT^ of PCR measurements (n= 6-13). For group definition, see Table 1 legends. TLR, toll-like receptor; MyD88, myeloid differentiation primary response protein 88; NF-κB, nuclear transcript factor kappa B; TNF-α, tumor necrosis factor α; IL, interleukin; TF, tissue factor; Ang, angiopoietin; SP, surfactant protein; IGF, insulin-like growth factor; VEGF, vascular endothelial GF; KGF, keratinocyte GF; AQP-5, aquaporin-5; eNOS, endothelial nitric oxide synthase; iNOS, inducible nitric oxide synthase. ^§^*p* < 0.05 vs. M. ^*^*p* < 0.05, ^**^*p* < 0.01, ^***^*p* < 0.001 vs. corresponding pooled non-designated drug group.

**S9 Table. Cox regression for survival time in ventilated newborn rabbits.**

___________________________________________________________________________

Factor aHR 95% CI *p*

___________________________________________________________________________

A. All animals (n = 184)

Group

M 1 (ref)

C 0.000 0.000- 0.977

MS 0.232 0.056-0.962 0.044

MH 0.060 0.007-0.552 0.013

MSH 0.115 0.013-0.976 0.047

MN 0.146 0.018-1.222 0.076

MSN 0.457 0.113-1.850 0.273

MHN 0.084 0.009-0.750 0.027

MSHN 0.000 0.000- 0.977

BW, g 1.039 0.991-1.089 0.113

PTX 27.546 6.808-111.449 0.000

___________________________________________________________________________

B. Animals for measuring Cdyn_mean_ (n = 178)

Cdyn_mean_ 0.000 0.000-0.015 0.001

___________________________________________________________________________

C. Animals allocated for histopathological measurement (n = 72)

LIS_total_ 1.485 0.866-2.547 0.151

___________________________________________________________________________

In A, all ventilated animals were involved. In B and C, only the animals allocated for Cdyn_mean_ and histopathological measurement were involved in the analysis, respectively. Group, BW and PTX listed in A are also included as independent variables in the regression model in B and C. aHR, adjusted hazard ratio. For group definitions in A, see Table 1 legends. BW, birthweight; Cdyn_mean_, the average Cdyn value during ventilation after drug administration; LIS_total_, total lung injury score.
