# Supplementary material for: Efficacy of heparin in respiratory support of near-term rabbits with meconium-induced acute lung injury: Linear regression model analyses
Source: PLoS One. 2026 Mar 24;21(3):e0345718. doi: 10.1371/journal.pone.0345718 (PMC13012467; doi:10.1371/journal.pone.0345718)
Supplement: S10 Table — (DOCX) [file pone.0345718.s002.docx]

**S10.1 Table. Overall model effect tests in** **LIS_total_**

_____________________________________________________________________

Effect type Variable/Interaction Likelihood ratio χ² df *p*

_____________________________________________________________________

Intercept 174.851 1 <0.001

Main effects PS 5.868 1 0.015

UFH 1.757 1 0.185

NO 32.314 1 <0.001

2-way interactions PS × UFH 1.757 1 0.185

PS × NO 5.033 1 0.025

UFH × NO 5.033 1 0.025

3-way interaction PS × UFH × NO 0.579 1 0.447

_____________________________________________________________________

**S10.2 Table. Parameter estimates in LIS_total_**

_____________________________________________________________________

Parameter combination B SE 95% CI *p*

(Coefficient)

_____________________________________________________________________

Intercept 7.75 0.464 [6.828, 8.672] <0.001

PS=1 (vs. PS=0) -2.25 0.656 [-3.554, -0.946] 0.001

UFH=1 (vs. UFH=0) -1.875 0.656 [-3.179, -0.571] 0.004

NO=1 (vs. NO=0) -3.875 0.656 [-5.179, -2.571] <0.001

PS=1 × UFH=1 1.375 0.927 [-0.470, 3.220] 0.138

PS=1 × NO=1 2 0.927 [0.155, 3.845] 0.031

UFH=1 × NO=1 2 0.927 [0.155, 3.845] 0.031

PS=1 × UFH=1 × NO=1 -1 1.311 [-3.609, 1.609] 0.446

_____________________________________________________________________

SE = standard error; CI = confidence interval. Reference groups: PS = 0, UFH = 0, NO = 0. Parameter estimates were obtained based on a generalized linear model analysis.

**S10.3 Table. Key pairwise comparisons of marginal mean differences in LIS_total_**

____________________________________________________________________

Mean

Group comparison difference SE 95% CI *p*

____________________________________________________________________

MSHN vs. MSN 0.50 0.656 [-1.23, -2.23] 1.000

MSHN vs. MHN 0.13 0.656 [-1.60, 1.85] 1.000

MSHN vs. MSH -0.87 0.656 [-2.60, 0.85] 1.000

MSHN vs. MH -1.75 0.656 [-3.48, -0.02] 0.046

MSN vs. MS -1.87 0.656 [-3.60, -0.15] 0.025

MHN vs. MH -1.87 0.656 [-3.60, -0.15] 0.025

MN vs. MH -2.00 0.656 [-3.73, -0.27] 0.014

MSN vs. M -4.12 0.656 [-5.85, -2.40] 0.001

MSH vs. M -2.75 0.656 [-4.48, -1.02] <0.001

MHN vs. M -3.75 0.656 [-5.48, -2.02] <0.001

MS vs. M -2.25 0.656 [-3.98, -0.52] 0.004

MH vs. M -1.87 0.656 [-3.60, -0.15] 0.025

MN vs. M -3.87 0.656 [-5.60, -2.15] <0.001

_____________________________________________________________________

PS and NO each exhibited significant independent main effects on the LIS_total_, both resulting in a pronounced reduction of the LIS_total_. However, significant negative interactions were observed for both NO×PS and NO×UFH, indicating that their combined effects were sub-additive (i.e., less than the sum of their individual effects). NO alone produced the most potent reduction in the LIS_total_. Comparisons of marginal means revealed that the concurrent administration of PS and NO (MSN group) or UFH and NO (MHN group) yielded a lower LIS_total_ than the administration of UFH and PS alone.

**S10.4 Table. Overall model effect tests in Vv**

_____________________________________________________________________

Effect type Variable/Interaction Likelihood ratio χ² df *p*

_____________________________________________________________________

Intercept 329.442 1 <0.001

Main effects PS 18.424 1 <0.001

UFH 5.868 1 0.015

NO 39.736 1 <0.001

2-way interactions PS × UFH 5.952 1 0.015

PS × NO 0.066 1 0.797

UFH × NO 1.434 1 0.231

3-way interaction PS × UFH × NO 2.569 1 0.109

_____________________________________________________________________

**S10.5 Table. Parameter estimates in Vv**

_____________________________________________________________________

Parameter combination B SE 95% CI *p*

(Coefficient)

_____________________________________________________________________

Intercept 0.494 0.0164 [0.461, 0.526] <0.001

PS=1 (vs. PS=0) 0.098 0.0232 [0.052, 0.145] <0.001

UFH=1 (vs. UFH=0) 0.091 0.0232 [0.044, 0.137] <0.001

NO=1 (vs. NO=0) 0.116 0.0232 [0.070, 0.162] <0.001

PS=1 × UFH=1 -0.096 0.0328 [-0.161, -0.030] 0.004

PS=1 × NO=1 -0.032 0.0328 [-0.097, 0.034] 0.336

UFH=1 × NO=1 -0.066 0.0328 [-0.131, 0.000] 0.046

PS=1 × UFH=1 × NO=1 0.075 0.0464 [-0.017, 0.168] 0.105

_____________________________________________________________________

SE = standard error; CI = confidence interval. Reference groups: PS = 0, UFH = 0, NO = 0. Parameter estimates were obtained based on a generalized linear model analysis.

**S10.6 Table. Key pairwise comparisons of marginal mean differences in Vv**

____________________________________________________________________

Group comparison Mean SE 95% CI *p*

difference

____________________________________________________________________

MSHN vs. MSH 0.094 0.0232 [0.033, 0.155] <0.001

MSHN vs. MH 0.097 0.0232 [0.036, 0.158] <0.001

MSHN vs. MS 0.089 0.0232 [0.028,0.134] 0.001

MSHN vs. MN 0.071 0.0232 [0.010, 0.150] 0.013

MSN vs. MSH 0.089 0.0232 [0.028, 0.151] 0.001

MSN vs. MN 0.067 0.0232 [0.006,0.128] 0.024

MSN vs. MS 0.084 0.0232 [0.023, 0.146] 0.002

MSN vs. M 0.183 0.0232 [0.122, 0.244] 0.001

MSH vs. M 0.093 0.0232 [0.032, 0.155] <0.001

MHN vs. M 0.141 0.0232 [0.080, 0.202] 0.001

MS vs. M 0.098 0.0232 [0.037, 0.160] <0.001

MH vs. M 0.091 0.0232 [0.029, 0.152] 0.001

MN vs. M 0.116 0.0232 [0.055, 0.177] 0.000

_____________________________________________________________________

Note: Only pairs that show statistical significance are listed.

Generalized linear model analysis revealed that the main effects of PS, UFH, and NO all had significant positive influences on Vv. Regarding interaction effects, a significant negative interaction was observed between PS and UFH (B = -0.096, *p* = 0.004), indicating that their combined use resulted in a smaller increase in Vv than the sum of their individual effects. The overall test for the three-way interaction among PS, UFH, and NO did not reach statistical significance (*p* = 0.109). 

**S10.7 Table. Overall model effect tests in CV(Vv)**

_____________________________________________________________________

Effect type Variable/Interaction Likelihood ratio χ² df *p*

_____________________________________________________________________

Intercept 216.189 1 <0.001

Main effects PS 11.229 1 0.001

UFH 4.123 1 0.042

NO 32.946 1 <0.001

2-way interactions PS × UFH 1.000 1 0.317

PS × NO 0.003 1 0.956

UFH × NO 0.952 1 0.329

3-way interaction PS × UFH × NO 2.122 1 0.145

_____________________________________________________________________

**S10.8 Table. Parameter estimates in CV(Vv)**

_____________________________________________________________________

Parameter combination B SE 95% CI *p*

(Coefficient)

_____________________________________________________________________

Intercept 0.293 0.014 (0.264, 0.321) <0.001

PS=1 (vs. PS=0) -0.061 0.020 (-0.101, -0.021) 0.003

UFH=1 (vs. UFH=0) -0.055 0.020 (-0.096, -0.015) 0.006

NO=1 (vs. NO=0) -0.091 0.020 (-0.131, -0.051) <0.001

PS=1 × UFH=1 0.050 0.029 (-0.007, 0.106) 0.080

PS=1 × NO=1 0.031 0.029 (-0.026, 0.087) 0.281

UFH=1 × NO=1 0.049 0.029 (-0.007, 0.106) 0.083

PS=1 × UFH=1 × NO=1 -0.059 0.040 (-0.139, 0.021) 0.142

_____________________________________________________________________

SE = standard error; CI = confidence interval. Reference groups: PS = 0, UFH = 0, NO = 0. Parameter estimates were obtained based on a generalized linear model analysis.

It reveals that PS, UFH, and NO all had significant and independent effects in reducing the dependent variable, CV(Vv) (as indicated by negative B coefficients). None of the two-way interactions (PS×UFH, PS×NO, UFH×NO) or the three-way interaction (PS×UFH×NO) were significant. This indicates that the effects of these three factors on CV**(**Vv) were primarily independent and additive, with no evidence of mutual moderation. As there were no significant differences among the groups for 2-way or 3-way interactive effects (S10.7 Table), herein pairwise comparisons of marginal mean differences in CV (Vv) are not provided.

**S10.9 Table. Overall model effect tests in pH**

_____________________________________________________________________

Effect type Variable/Interaction Likelihood ratio χ² df *p*

_____________________________________________________________________

Intercept 1063.445 1 < 0.001

Main effects PS 13.442 1 < 0.001

UFH 0.042 1 0.837

NO 28.772 1 < 0.001

2-way interactions PS × UFH 4.273 1 0.039

PS × NO 2.208 1 0.137

UFH × NO 2.974 1 0.085

3-way interaction PS × UFH × NO 6.052 1 0.014

_____________________________________________________________________

**S10.10 Table. Parameter estimates in pH**

_____________________________________________________________________

Parameter combination B SE 95% CI *p*

(Coefficient)

_____________________________________________________________________

Intercept 7.134 0.0334 [7.068, 7.200] < 0.001

PS=1 (vs. PS=0) 0.188 0.0431 [0.103, 0.273] < 0.001

UFH=1 (vs. UFH=0) 0.125 0.0421 [0.041, 0.208] 0.003

NO=1 (vs. NO=0) 0.218 0.0426 [0.134, 0.302] < 0.001

PS=1 × UFH=1 -0.175 0.0560 [-0.286, -0.064] 0.002

PS=1 × NO=1 -0.152 0.0574 [-0.266, -0.039] 0.008

UFH=1 × NO=1 -0.162 0.0559 [-0.272, -0.051] 0.004

PS=1 × UFH=1 × NO=1 0.190 0.0765 [0.039, 0.342] 0.013

_____________________________________________________________________

SE = standard error; CI = confidence interval. Reference groups: PS = 0, UFH = 0, NO = 0. Parameter estimates were obtained based on a generalized linear model analysis.

**S10.11 Table. Key pairwise comparisons of marginal mean differences in pH**

____________________________________________________________________

Group comparison Mean SE 95% CI *p*

difference

____________________________________________________________________

MSHN vs. MSH 0.095 0.035 [0.002 0.187] 0.043

MSHN vs. MH 0.108 0.036 [0.013 0.202] 0.015

MSN vs. MSH 0.116 0.037 [0.019, 0.214] 0.010

MSN vs. M 0.254 0.043 [0.140, 0.368] <0.001

MSH vs. M 0.138 0.042 [0.028, 0.248] 0.006

MHN vs. M 0.181 0.042 [0.070, 0.292] <0.001

MS vs. M 0.188 0.043 [0.074, 0.302] <0.001

MH vs. M 0.125 0.042 [0.014, 0.236] 0.018

MN vs. M 0.218 0.043 [0.106, 0.330] <0.001

_____________________________________________________________________

This study revealed that the regulatory mechanisms of PS, UFH, and NO on pH are highly complex, involving a significant three-way interaction. Each factor individually elevated pH when present alone. However, when any two factors were combined, their effects were antagonistic, mutually attenuating the pH increase. Intriguingly, when all three factors coexisted, a synergistic or compensatory mechanism emerged, partially reversing the antagonism observed in two-factor combinations. This resulted in a final pH value higher than that predicted by a simple antagonistic model. For instance, when UFH was present (UFH = 1), the pH in the [PS = 1, NO = 1] group was significantly higher than that in the [PS = 1, NO = 0] group (*p* = 0.043, S10.11 Table, line 1). This indicates that in the presence of both PS and UFH, the addition of NO further increased the pH, exemplifying the manifestation of the three-way interaction.

**S10.12 Table. Overall model effect tests in PCO_2_**

_____________________________________________________________________

Effect type Variable/Interaction Likelihood ratio χ² df *p*

_____________________________________________________________________

Intercept 332.602 1 < 0.001

Main effects PS 11.219 1 0.001

UFH 0.098 1 0.755

NO 15.665 1 < 0.001

2-way interactions PS × UFH 0.473 1 0.491

PS × NO 0.741 1 0.389

UFH × NO 1.551 1 0.213

3-way interaction PS × UFH × NO 3.084 1 0.079

_____________________________________________________________________

**S10.13 Table. Parameter estimates in** **PCO_2_**

_____________________________________________________________________

Parameter combination B SE 95% CI *p*

(Coefficient)

_____________________________________________________________________

Intercept 83.67 5.59 [72.620, 94.720] < 0.001

PS=1 (vs. PS=0) -21.60 7.22 [-35.860, -7.340] 0.003

UFH=1 (vs. UFH=0) -10.864 7.05 [-24.786, 3.057] 0.123

NO=1 (vs. NO=0) -25.50 7.13 [-39.580, -11.420] < 0.001

PS=1 × UFH=1 15.730 9.38 [-2.789, 34.249] 0.093

PS=1 × NO=1 16.841 9.62 [-2.159, 35.841] 0.080

UFH=1 × NO=1 19.319 9.36 [0.830, 37.808] 0.039

PS=1 × UFH=1 × NO=1 -22.637 12.81 [-47.938, 2.664] 0.077

_____________________________________________________________________

SE = standard error; CI = confidence interval. Reference groups: PS = 0, UFH = 0, NO = 0.

This study demonstrates that PS and NO are independent factors in reducing PCO₂. UFH had no significant main effect. No two-way or three-way interactions reached statistical significance. Therefore, pairwise comparisons of marginal mean differences in PCO_2_ are not provided.

It suggests that in a standardized analytical framework, conclusions should be drawn based on the overall model effects test (omnibus test). When the overall test is not statistically significant, interpretation of the significance of individual parameters within that term requires extreme caution and generally should not be relied upon for definitive inference.

**S10.14 Table. Overall model effect tests in LAC**

_____________________________________________________________________

Effect type Variable/Interaction Likelihood ratio χ² df *p*

_____________________________________________________________________

Intercept 206.327 1 < 0.001

Main effects PS 3.909 1 0.048

UFH 2.225 1 0.136

NO 0.905 1 0.341

2-way interactions PS × UFH 1.917 1 0.166

PS × NO 3.576 1 0.059

UFH × NO 3.467 1 0.063

3-way interaction PS × UFH × NO 1.353 1 0.245

_____________________________________________________________________

**S10.15 Table. Parameter estimates in LAC**

_____________________________________________________________________

Parameter combination B SE 95% CI *p*

(Coefficient)

_____________________________________________________________________

Intercept 6.470 0.672 [5.143, 7.797] <0.001

PS=1 (vs. PS=0) -2.483 0.868 [-4.197, -0.770] 0.004

UFH=1 (vs. UFH=0) -2.282 0.847 [-3.955, -0.609] 0.007

NO=1 (vs. NO=0) -2.270 0.857 [-3.962, -0.578] 0.008

PS=1 × UFH=1 1.967 1.1267 [-0.258, 4.193] 0.081

PS=1 × NO=1 2.363 1.156 [0.080, 4.646] 0.041

UFH=1 × NO=1 2.341 1.125 [0.119, 4.562] 0.037

PS=1 × UFH=1 × NO=1 -1.795 1.5393 [-4.835, 1.245] 0.244

_____________________________________________________________________

SE = standard error; CI = confidence interval. Reference groups: PS = 0, UFH = 0, NO = 0.

According to the model effects test (likelihood ratio test), only PS had a significant independent main effect on LAC (*p* = 0.048). The main effects of UFH and NO, along with all two-way and three-way interactions, did not reach statistical significance in the overall model test. Therefore, pairwise comparisons of marginal mean differences in LAC are not provided.

The parameter estimates table indicated significant differences for the main effects of UFH and NO, as well as for the PS×NO and UFH×NO interaction terms (*p* < 0.05). However, because their corresponding overall model effects tests were not statistically significant, these findings must be interpreted with extreme caution and should not be considered the primary conclusions of this study. Therefore, this model concluded that PS had a significant reducing effect on LAC. The suggestive findings regarding UFH, NO, and their interactions require validation in larger samples.

**S10.16 Table. Overall model effect tests in DSPC_BALF_**

_____________________________________________________________________

Effect type Variable/Interaction Likelihood ratio χ² df *p*

_____________________________________________________________________

Intercept 146.248 1 < 0.001

Main effects PS 25.062 1 < 0.001

UFH 0.000 1 0.996

NO 0.214 1 0.644

2-way interactions PS × UFH 0.735 1 0.391

PS × NO 0.016 1 0.901

UFH × NO 0.162 1 0.687

3-way interaction PS × UFH × NO 2.895 1 0.089

_____________________________________________________________________

**S10.17 Table. Parameter estimates in DSPC_BALF_**

_____________________________________________________________________

Parameter combination B SE 95% CI *p*

(Coefficient)

_____________________________________________________________________

Intercept 0.552 0.141 [0.273, 0.832] < 0.001

PS=1 (vs. PS=0) 0.823 0.204 [0.420, 1.227] < 0.001

UFH=1 (vs. UFH=0) 0.221 0.209 [-0.193, 0.635] 0.290

NO=1 (vs. NO=0) 0.194 0.200 [-0.201, 0.589] 0.331

PS=1 × UFH=1 -0.526 0.295 [-1.110, 0.059] 0.075

PS=1 × NO=1 -0.376 0.288 [-0.946, 0.195] 0.192

UFH=1 × NO=1 -0.268 0.289 [-0.840, 0.304] 0.354

PS=1 × UFH=1 × NO=1 0.700 0.408 [-0.108, 1.509] 0.086

_____________________________________________________________________

SE = standard error; CI = confidence interval. Reference groups: PS = 0, UFH = 0, NO = 0.

Generalized linear model analysis indicated that, after adjusting for other factors, only PS had a significant independent main effect on DSPC_BALF_ levels (Likelihood ratio Chi-Square = 25.062, *p* < 0.001). The presence of PS was associated with a significant increase in DSPC_BALF_ levels (B = 0.823, 95% CI: 0.420 to 1.227). The main effects of UFH and NO, as well as all two-way interactions (PS×UFH, PS×NO, UFH×NO) and the PS×UFH×NO three-way interaction, did not reach statistical significance in the overall model test (all *p* > 0.05).

**S10.18 Table. Overall model effect tests in survival outcome**

_____________________________________________________________________

Effect type Variable/Interaction Likelihood ratio χ² df *p*

_____________________________________________________________________

Intercept 126.937 1 < 0.001

Main effects PS 0.794 1 0.373

UFH 6.352 1 0.012

NO 2.742 1 0.098

2-way interactions PS × UFH 0.977 1 0.323

PS × NO 0.178 1 0.673

UFH × NO 0.194 1 0.659

3-way interaction PS × UFH × NO 2.635 1 0.105

_____________________________________________________________________

**S10.19 Table. Parameter estimates in survival outcome**

_____________________________________________________________________

Parameter combination B SE OR 95% CI *p*

_____________________________________________________________________

Intercept -0.693 0.463 0.500 [0.190, 1.202] 0.134

PS=1 (vs. PS=0) -1.041 0.779 0.353 [0.066, 1.527] 0.181

UFH=1 (vs. UFH=0) -2.251 1.126 0.105 [0.005, 0.687] 0.045

NO=1 (vs. NO=0) -2.251 1.126 0.105 [0.005, 0.687] 0.045

PS=1 × UFH=1 1.041 1.647 2.833 [0.082, 100.5] 0.527

PS=1 × NO=1 2.251 1.432 9.500 [0.687, 271.7] 0.116

UFH=1 × NO=1 2.200 1.836 9.025 [0.211, 525.7] 0.231

PS=1 × UFH=1 × NO=1 -21.822 17340 - [0.000, 0.000] 0.999

_____________________________________________________________________

SE = standard error; CI = confidence interval. Reference groups: PS = 0, UFH = 0, NO = 0.

Generalized linear regression analysis (dependent variable: 0 = survival, 1 = death) revealed that, after adjusting for other factors, only UFH had a significant independent main effect on mortality risk (Likelihood ratio Chi-square = 6.352, *p* = 0.012, S10.18 Table). The use of UFH (UFH = 1) was a significant protective factor, associated with a markedly reduced mortality risk. The odds ratio (OR) was 0.105 (95% CI: 0.005 to 0.687). PS had no significant effect on mortality risk (*p* = 0.373). NO showed a potential protective trend (OR = 0.105), but it did not reach statistical significance in the overall model test (*p* = 0.098, S10.18 Table); this finding requires cautious interpretation. All two-way and three-way interactions were not statistically significant in the overall model test (all *p* > 0.05). The parameter estimate for the PS×UFH×NO three-way interaction was anomalous (B = -21.822, with an extremely large standard error), a result of "complete separation" in the data, rendering this estimate devoid of practical meaning.

**S10.20 Table. Overall model effect tests in W/D**

_____________________________________________________________________

Effect Type Variable/Interaction Likelihood ratio χ² df *p*

_____________________________________________________________________

Intercept 546.710 1 < 0.001

Main effects PS 0.320 1 0.572

UFH 3.732 1 0.053

NO 4.405 1 0.036

2-way interactions PS × UFH 1.003 1 0.317

PS × NO 0.407 1 0.524

UFH × NO 0.296 1 0.586

3-way interaction PS × UFH × NO 3.652 1 0.056

_____________________________________________________________________

**S10.21 Table. Parameter estimates in W/D**

_____________________________________________________________________

Parameter combination B SE 95% CI *p*

(Coefficient)

_____________________________________________________________________

Intercept 6.206 0.219 [5.775, 6.638] < 0.001

PS=1 (vs. PS=0) -0.331 0.310 [-0.941, 0.280] 0.285

UFH=1 (vs. UFH=0) -0.361 0.310 [-0.971, 0.249] 0.243

NO=1 (vs. NO=0) -0.643 0.314 [-1.262, -0.024] 0.040

PS=1 × UFH=1 0.286 0.438 [-0.577, 1.150] 0.513

PS=1 × NO=1 0.797 0.438 [-0.067, 1.661] 0.069

UFH=1 × NO=1 0.429 0.436 [-0.430, 1.288] 0.324

PS=1 × UFH=1 × NO=1 -1.197 0.623 [-2.425, 0.031] 0.055

_____________________________________________________________________

SE = standard error; CI = confidence interval. Reference groups: PS = 0, UFH = 0, NO = 0.

Generalized linear model analysis indicated that, after adjusting for other factors, only NO had a significant independent main effect on W/D levels (Likelihood ratio Chi-square = 4.405, *p* = 0.036). The presence of NO (NO = 1) was associated with a significant reduction in W/D levels (B = -0.643, 95% CI: -1.262 to -0.024). The main effects of UFH and PS did not reach statistical significance (*p* > 0.05). All two-way interactions (PS×UFH, PS×NO, UFH×NO) as well as the PS×UFH×NO three-way interaction were not statistically significant in the overall model test (all *p* > 0.05).

**S10.22 Table. Overall model effect tests in Cdyn_mean_**

_____________________________________________________________________

Effect Type Variable/Interaction Likelihood ratio χ² df *p*

_____________________________________________________________________

Intercept 384.633 1 <0.001

Main effects PS 0.211 1 0.646

UFH 2.170 1 0.141

NO 3.830 1 0.050

2-way interactions PS × UFH 4.092 1 0.043

PS × NO 2.634 1 0.105

UFH × NO 2.201 1 0.138

3-way interaction PS × UFH × NO 3.194 1 0.074

_____________________________________________________________________

**S10.23 Table. Parameter estimates in Cdyn_mean_**

_____________________________________________________________________

Parameter combination B SE 95% CI *p*

(Coefficient)

_____________________________________________________________________

Intercept 0.514 0.054 (0.408, 0.620) < 0.001

PS=1 (vs. PS=0) 0.204 0.072 (0.062, 0.346) 0.005

UFH=1 (vs. UFH=0) 0.234 0.072 (0.092, 0.376) 0.001

NO=1 (vs. NO=0) 0.237 0.073 (0.093, 0.380) 0.001

PS=1 × UFH=1 -0.264 0.099 (-0.459, -0.068) 0.008

PS=1 × NO=1 -0.236 0.100 (-0.432, -0.039) 0.018

UFH=1 × NO=1 -0.226 0.099 (-0.421, -0.031) 0.023

PS=1 × UFH=1 × NO=1 0.247 0.138 (-0.024, 0.518) 0.072

_____________________________________________________________________

**S10.24 Table. Key pairwise comparisons of marginal mean differences in Cdyn_mean_**

____________________________________________________________________

Group comparison Mean SE 95% CI p-value

difference

____________________________________________________________________

MSN vs. M 0.205 0.072 [0.015, 0.395] 0.027

MHN vs. M 0.245 0.071 [0.057, 0.433] 0.004

MS vs. M 0.204 0.072 [0.014, 0.394] 0.028

MH vs. M 0.234 0.072 [0.044, 0.424] 0.007

MN vs. M 0.237 0.073 [0.045, 0.429] 0.007

_____________________________________________________________________

When significant interaction terms are present, the interpretation of main effects requires great caution. Their "non-significance" may be due to the effects being masked or moderated by the interactions. Generalized linear model analysis revealed that PS, UFH, and NO each independently improved mean dynamic lung compliance (Cdyn_mean_). However, a significantly negative interaction effect was observed between PS and UFH, indicating that their combined benefit was lower than expected. The modulating effect of NO on Cdyn_mean_ was substantial: in the absence of NO, the independent improvement effects of PS or UFH were clearly evident; however, adding NO or altering the status of the other drug did not provide significant additional gains in Cdyn_mean_. This reaffirms that the effect of NO may be "masked" or "saturated" by the presence of PS or UFH. The complex higher-order interactions among the three factors warrant further investigation in future studies.

Note: Cdyn_mean_, an average of Cdyn values over the last 7 hours of 10-h ventilation.
