# Supplementary figures and images for: Efficacy of heparin in respiratory support of near-term rabbits with meconium-induced acute lung injury: Linear regression model analyses
Source: PLoS One. 2026 Mar 24;21(3):e0345718. doi: 10.1371/journal.pone.0345718 (PMC13012467; doi:10.1371/journal.pone.0345718)

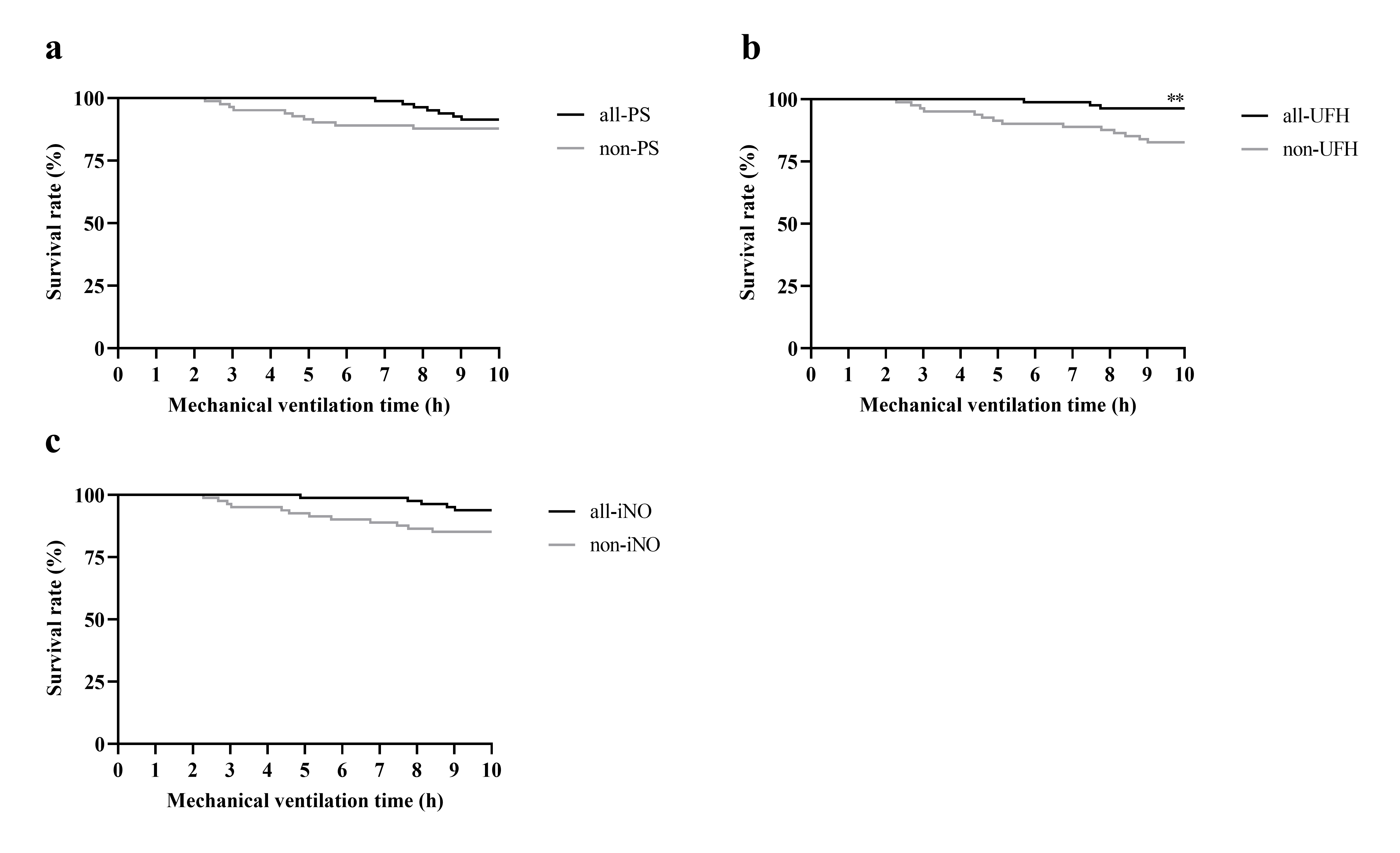

Supplement: S1 Fig — **p < 0.01 vs. corresponding pooled non-designated drug group by log-rank test. For group definition, see Table 1 legends (n = 81–82). (TIF) [file pone.0345718.s003.tif]

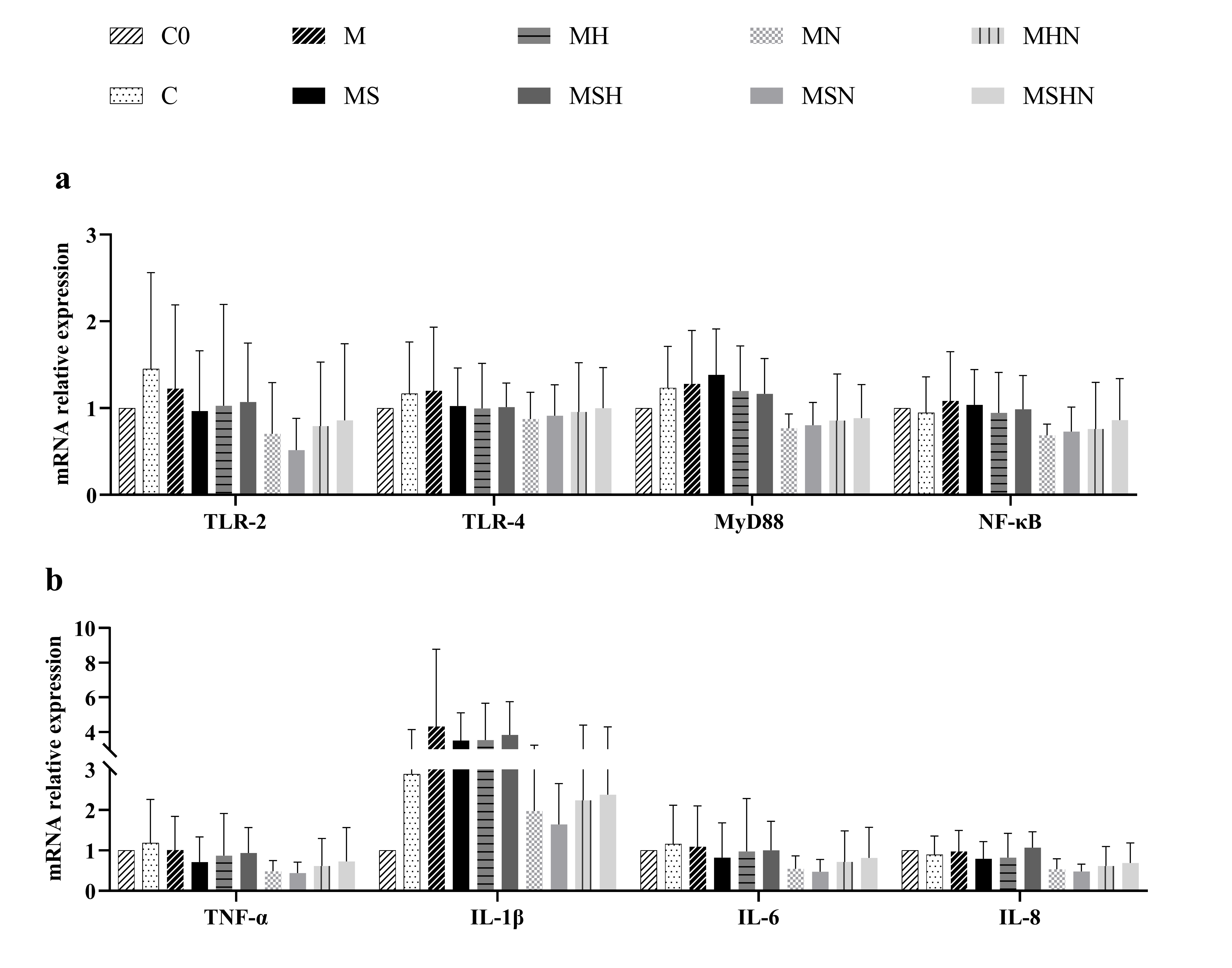

Supplement: S2 Fig — Values are expressed as means and SD of 2−ΔΔCT of PCR measurements (n = 8–13). For group definition, see Table 1 legends. TLR, toll-like receptor; MyD88, myeloid differentiation primary response protein 88; NF-κB, nuclear transcript factor kappa B; TNF-α, tumor necrosis factor α; IL, interleukin. (TIF) [file pone.0345718.s004.tif]

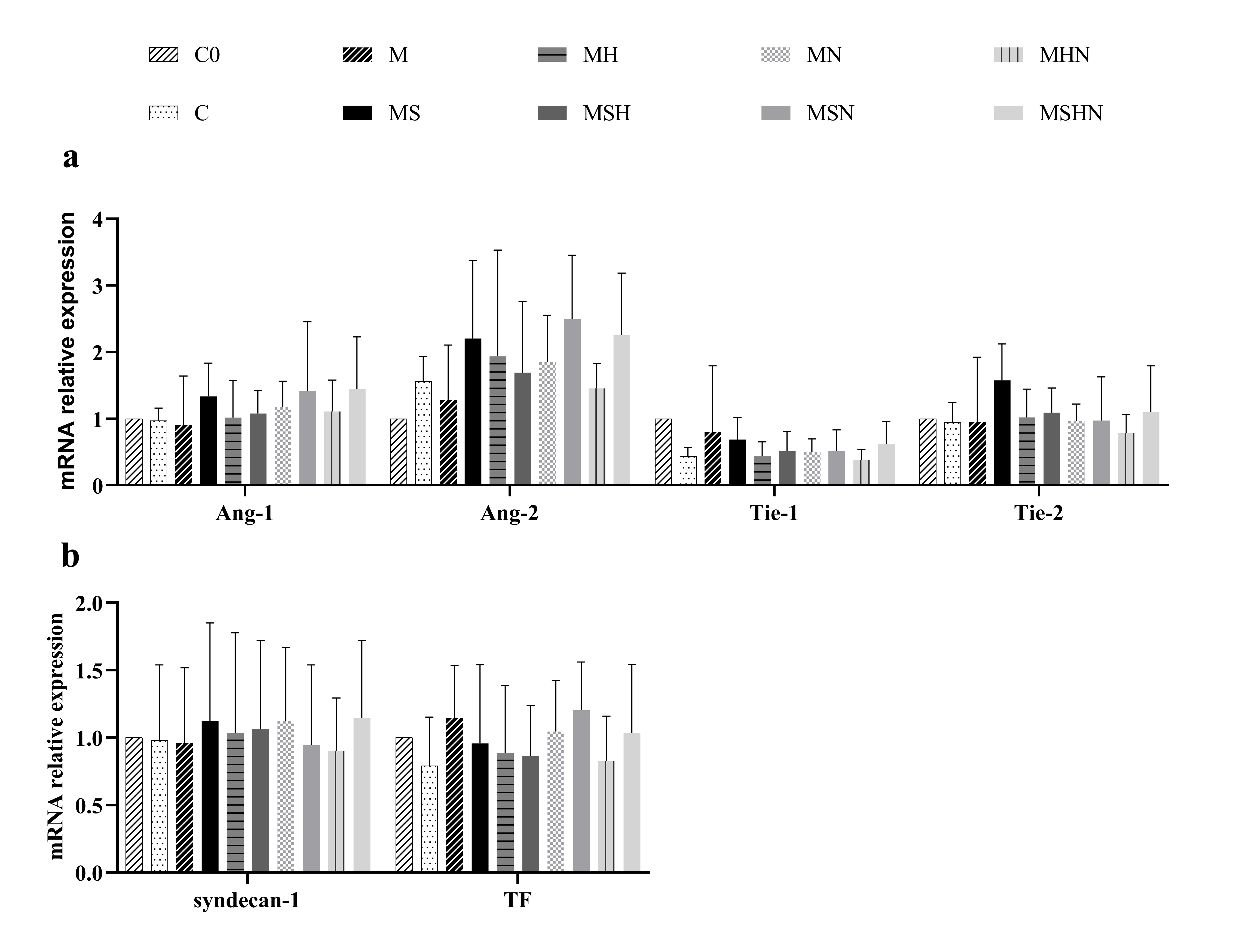

Supplement: S3 Fig — Values are expressed as means and SD of 2−ΔΔCT of PCR measurements (n = 6–13). For group definition, see Table 1 legends. Ang, angiopoietin; TF, tissue factor. (TIF) [file pone.0345718.s005.tif]

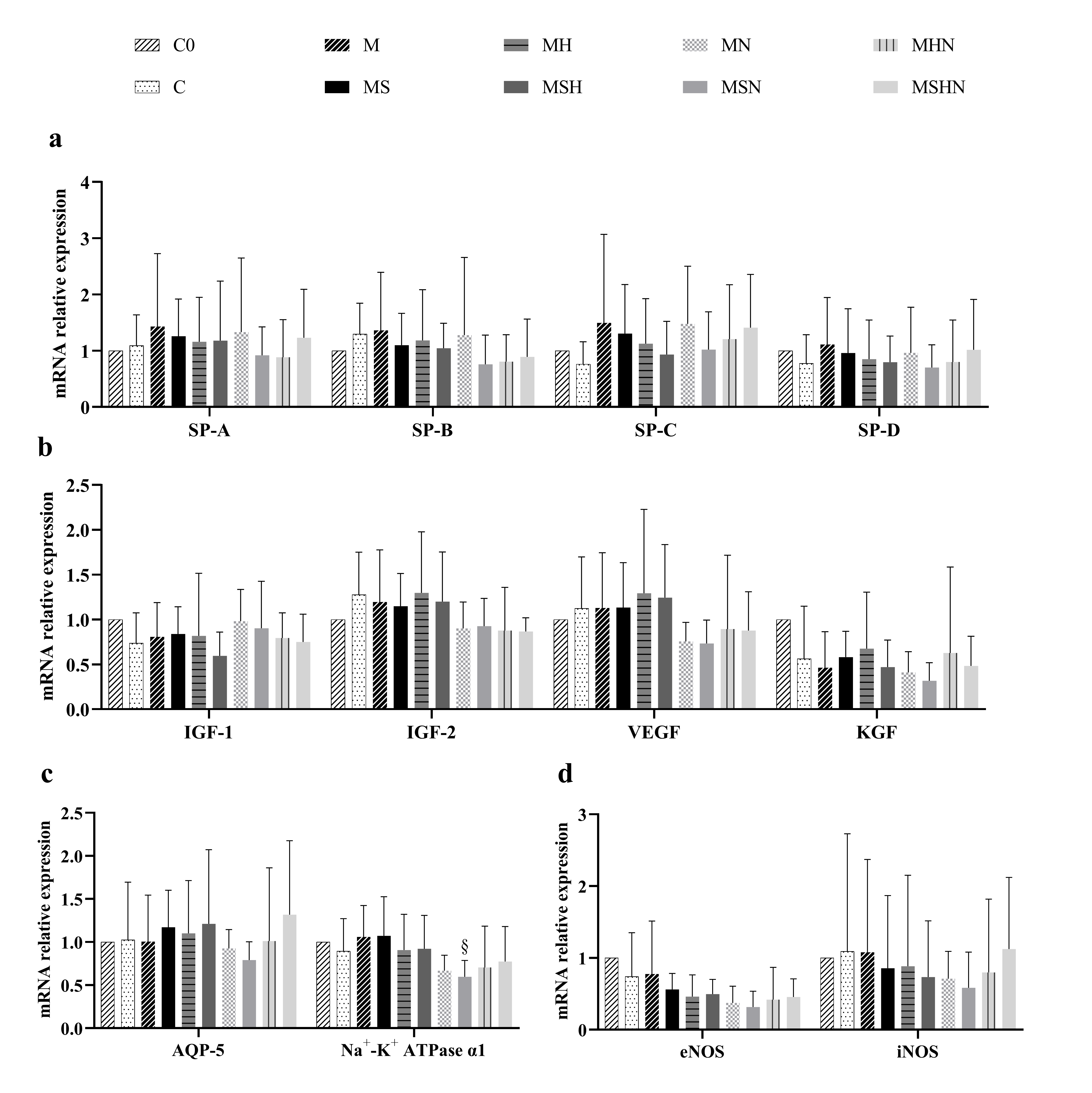

Supplement: S4 Fig — Values are expressed as means and SD of 2−ΔΔCT of PCR measurements (n = 8−13). For group definition, see Table 1 legends. SP, surfactant protein; IGF, insulin-like growth factor; VEGF, vascular endothelial GF; KGF, keratinocyte GF; AQP-5, aquaporin-5; eNOS, endothelial nitric oxide synthase; iNOS, inducible nitric oxide synthase. §p < 0.05 vs. M. (TIF) [file pone.0345718.s006.tif]
