# Supplementary material for: Efficacy of heparin in respiratory support of near-term rabbits with meconium-induced acute lung injury: Linear regression model analyses
Source: PLoS One. 2026 Mar 24;21(3):e0345718. doi: 10.1371/journal.pone.0345718 (PMC13012467; doi:10.1371/journal.pone.0345718)
Supplement: Attachment — Submitted filename: Response to reviewers-20251015.docx [file pone.0345718.s009.docx]

Response to Reviewers'	20251015

We are very grateful to the reviewers for their comments regarding the improvement of our manuscript. We have made thorough revision according to the points raised by both reviewers and academic editor, and summarized response point-by-point below, to the queries (Qx) in order of the decision letter of 20250901. In the current version (R1), yellow highlights indicate new or modified content, while red words/fonts denote deleted content.

Review Comments to the Author

Reviewer #1:

**Q1**. In this study, Xie et al investigate the effects of heparin in near term newborn rabbits in a model of mechanical and chemical induced lung injury in an effort to identify therapeutic options for meconium aspiration syndrome. I commend the authors on using appropriate controls for the various experiments.

**Response:**

Thanks for the comments. Regarding the control groups, our preliminary design was to evaluate, in an established near-term newborn rabbit MAS model, the efficacy of heparin, either standing alone or in combination with surfactant and/or inhaled NO. The controls were in two groups, either not receiving meconium (group C), or given meconium but no additional specific medications (group M), to undergo standardized tidal volume ventilation and to set best and worst survival limit, as shown by Kaplan-Meier survival curves (Fig. 2). This enabled evaluation of the therapeutic action of specific respiratory medications, hence explore the underlying mechanisms. There was a technical limitation for the groups related to exposure to iNO as there was no additional multi-plethysmograph-ventilator to ensure intra-litter randomization. Therefore, in the second phase of the experiment, groups were all exposed to iNO through a mixed inter- and intra-litter randomization, in which heparin and PS were randomized within litters, thus comparable to those in the non-iNO groups in randomization. In both phases, the ventilation settings and other medications were similar following the experimental protocols. In the marked R1, on page 14, lines 289–292, we have modified the description of statistic tests for the groups in two subsets: one by using group M and C as controls, and three groups without NO (MS, MH, MSH); and the other, the four groups with NO (MN, MSN, MHN, MSHN) were compared separately versus the group C and M. One-way ANOVA (F test) was used with post hoc test for repeated measures. There was no direct comparison of the two corresponding treatment groups across the two sets (i.e., MH vs. MHN, MS vs. MSN, and MSH vs. MSHN, respectively). As the mean values, SD and sample size are available, readers should be able to readily estimate the differences in-between.

Surfactant is off-label used in daily practice in NICU for late preterm and term/post-term newborn infants at risk of respiratory distress due to meconium aspiration, while iNO is applied for persistent hypoxemic respiratory failure complicated with persistent pulmonary hypertension (PPHN).

I have the following concerns:
Q2. - One important issue with heparin is that is also known to bind to angiogenic factors and can inhibit late stage alveolar development. There are several studies that demonstrate that heparin inhibits lung growth and can potentiate lung injury as a result of this mechanism. The authors should consider this additional data especially in light of their own findings. This concern should be addressed in a revised version of the manuscript. For example angiogenic factors such as VEGF and VEGFR activation can be evaluated in lung samples.

**Response:**

Thanks for the concern about long-lasting adverse effects of heparin on the angiogenesis of neonatal lungs. As proposed, in the marked R1, on page 5, lines 101–109, we read the reference (ref. 23 in R1) about heparin hindering late alveolar development in a mature mouse model. Their dosage of heparin was several times higher than what we provided, and their conclusions may not be generalizable considering our peculiar experimental design and overall and specific efficacies, and possible half-life effect of heparin. We also performed additional examination by PCR for lung tissue mRNA expression of Ang-1, Ang-2, and their receptors, Tie-1 and Tie-2, from the lung tissue samples of all the treatment groups. The data are included in supplemental information S3 Fig. and S8 Table of R1. There is no evidence to consider that the intrapulmonary vasculature was substantially impaired attributable to heparin and associated other medications.

The role of Ang-2 in angiogenesis remains unclear. It primarily exerts anti-angiogenic effects by inhibiting Ang-1-induced Tie-2 phosphorylation, while Tie-1 blocks the Ang-1 signaling pathway by inhibiting Tie-2. In an in vitro study, it indicates that in the absence of Ang-1, Ang-2 can act as a weak agonist for Tie-2, promoting phosphorylation of Tie-2, thereby inhibiting endothelial cell apoptosis and enhancing migration. (Yuan HT, Khankin EV et al. Mol Cell Biol. 2009, 29(8): 2011-22). Tie-1 and Tie-2 exist in hetero-oligomeric complexes on the endothelial surface, with Tie-1 inhibiting Tie-2. When VEGF levels transiently increase, it stimulates Tie-1 cleavage, thereby enhancing Ang-1 signaling. Furthermore, Ang-2 overexpression is associated with increase in vascular permeability and may be considered an inflammation-associated marker [ref. 56, 57 in R1]. Notably, mRNA expression of these molecules and mediators in this study may undergo a developmental change in magnitude as response to the intervention in early postnatal life of the experimental animals. For technical difficulty (lack of antibody against specific rabbit antigen), we did not measure their metabolites of mRNA expression (by ELISA or western blot), nor for their time course alteration. As mentioned above, it also requires specific study plan and protocol to verify.

Q3. - I do think that it is essential to consider measuring coagulation parameters in the groups that received heparin vs. not (e.g. Factor Xa assay vs. partial thromboplastin time measurement)
**Response:**

Thanks for this recommendation. In the original manuscript, we have reported mRNA expression of Syndecan-1, tissue factor (TF) and VEGF to connect the intrapulmonary vascular status, in part associated with coagulation as explained in the above response to Q2. Regarding the pharmacological action of anticoagulation by heparin, due to the very low blood volume accessible at the end of experiment for tiny pups (BW circ. 40-55 g), and to share it with other measurements (blood gas), it was not considered to measure blood clotting and coagulation by APTT or ACT. We examined hemorrhage in the lung injury score (LIS, items and total, Table 1 and S5 Table in R1) measurement. There was no tangible evidence of excessive bleeding in the lungs from those exposed to heparin. In the marked R1, on page 6, lines 112–118, we have added a reference for heparin use in neonates by the American Pharmacists Association (ref. 26 of R1), in which there was no prohibition or restriction in neonatal infants unless its use in ECMO, in which it should be subject to repeated measures of ACT for systemic heparinization. Considering topic use of heparin in indwelling catheterization at NICU practice, the adverse effects of heparin may be confined to routine clinical pharmacology surveillance. Studies evaluating the risk of UFH-related bleeding in the neonatal and pediatric populations are limited. A well-design experiment using relatively larger animal model should be expected to test the coagulation of heparin in this kind of MAS-ALI model to answer the query. And prospective studies are needed to determine the precise risk of UFH-associated bleeding. Nevertheless, the common cause of fatal UFH-induced bleeding is due to accidental overdose [ref. 41 of R1].

Q4. - An additional concern is the effect of lung injury appears to be minimal based on the lung injury scoring (Table 2). Is this similar to other models/expected? Can the authors comment? My concern would be is that the disease is not severe enough and therefore we cannot make any conclusions on whether or not heparin itself has an additive effect on MAS treatment or we are just seeing the well established effects of surfactant/iNO?

**Response:**

Thanks for this comment. We do not consider the lung injury severity should be minimal. The measurements of lung injury type and severity should consist of LIS and prevalence of pneumothorax (PTX). Other variables like Cdyn, W/D, DSPC/TP may add to understanding the injury pattern, magnitude, and underlying mechanisms for lung liquid clearance, surface tension vs. vascular-to-alveolar permeability, respectively. The meconium preparation, administration, and response pattern in current experiment are similar to our previous ones (Xu Y et al, Pediatr Res 2023, ref. 27 in R1). The major difference was the doubled concentration of meconium in suspension (100 mg/ml, 4 ml/kg) in current study so as to minimize fluid load, which was associated with the lower early deaths and PTX compared to the previous one [final survival rate 67% (14/21) vs. 26.5% (16/61); 10% (2/21) vs. 23% (14/61), respectively], along with reduced inflammatory response. This improvement may be attributed to the reduced viscosity of meconium suspension, which minimized large airway obstruction but retained underlying hypoxia-induced, hyperoxic stress-mediated, inflammatory response. [As a note, the previous MAS study (ref. 27) required additional meconium dose (1-3 mL/kg) in part of the animals in each group to achieve hypoxemic respiratory failure and lung injury before the commence of surfactant treatment. Thus, it incurs large fluid volume load to the lung mechanics, injury severity and overall survival differences]. The difference in current survival curve of all treated groups may also be related to more delicate management during ventilation, and the type of surfactant preparations [in current study as BALF-derived, containing higher phosphatidylcholine (PC) vs. lung tissue homogenate-derived, containing high phosphatidylethanolamine (PE) of non-type II cells, or synthetic peptide-phospholipids of selected phosphatidylcholine (PC) and phosphatidylglycerol (PG) in ref. 27] as well as the use of iNO and heparin.

Human meconium is bacteria-free, contains neutral lipids, and may not elicit infection. The pathophysiology of MAS is associated with systemic hypoxemia with lung exposed to hyperoxic stress. Therefore, the lung injury should be considered non-infectious. We relied on the measurement of lung mechanics, lung histopathology, morphometrics and LIS, surfactant phospholipid pools, and expression of mRNA in lung tissues representative of different epithelial and endothelial cell types, to characterize the lung injury type and severity, and the efficacy of heparin and its combination with PS and iNO. Different from preterm lungs, which are at late canalicular to early saccular stage of development, and prone to respiratory distress syndrome (RDS), the near-term rabbit lungs are at alveolar stage, with plenty of surfactant phospholipids and specific proteins (SP-A, B, C). Thus, the lung injury pattern should be characterized as in term neonatal lungs with sufficient surfactant pools, which are >10 times at birth that of the adult and children, with meconium-induced chemical alveolitis, partial airway obstruction, and impaired endogenous surfactant metabolism. This point is addressed in the Results and Discussion of R1.

In the marked R1, on page 12, lines 243–247, we have re-examined all the lung tissue sections for histopathology and performed LIS again by including meconium distribution in alveolar space and small airways as an additional item in the lung injury score (LIS). This is added in supplemental table S5 and Table 1 (in both marked and clean body text content of R1), and should more comprehensively account for the lung injury pattern. Based on the survival length and rate, lung mechanics, LIS, lung surfactant phospholipid pools, and integrated mRNA expression levels of relevant molecules of lung cells, the impact of heparin was discernible with PS and iNO as shown in S2-S9 Table.

Reviewer #2:

Thank you for the opportunity to review the manuscript entitled “Efficacy of heparin in respiratory support of near-term newborn rabbits with meconium-induced acute lung injury.” This study investigates whether adding heparin to standard therapy improves outcomes in a meconium aspiration-induced lung injury model.

The topic is interesting and clinically relevant; however, there are several critical issues that need to be addressed. In particular, the unsophisticated statistical approach—comparing multiple parameters across many groups—makes interpretation difficult. Furthermore, some results raise concerns about the validity of the acute lung injury model itself. My main concerns are as follows:

Q5. 1. Language quality – The manuscript contains numerous grammatical errors and unclear expressions. Language editing by a native English speaker is strongly recommended.
**Response:**

Thanks for the comments. We have extensively revised the manuscript using tools for spelling, writing and grammar. Long sentences are shortened, data presentation, interpretation, concept and points are expressed more succinctly, for comprehension. All the changes are marked in R1.

Q6. 2. Anesthesia and lidocaine use – Please clarify how the pups were anesthetized. Why was lidocaine added to the supplemental fluid? Also, it is unclear whether the administered dose was appropriate.
**Response:**

Thanks for the comments. The analgetic used for does in sequence of diazepam and urethane, along with a face mask and oxygen were to minimize the risk of hypoxemia-associated fetal stress during delivery. The newborn pups were taken out by C-section, dried and weighed. Initial anesthesia was administered via intraperitoneal injection of diluted lidocaine solution to facilitate intratracheal intubation. Subsequent lidocaine was given intraperitoneally with glucose and sodium bicarbonate diluted in different proportions for respective physiological need and relevance (lines 161-162 and 174-176, page 8, in marked R1). This initial anaesthetic procedure and protocol was adopted from the early experiments, in the 1980-1990 era, of short-time (30 min-5 hours) ventilation of rabbits at 27-30 days of gestational age (GA, term 31 days) at Karolinska experimental perinatal pathology laboratory (Dr. Bengt Robertson), Stockholm, Sweden. For peritoneal intermittent injection, it also followed our previous experience with ventilated very preterm rabbit pups (GA 26 days) studies, which provided glucose, sodium bicarbonate and lidocaine for energy, counter balance acidosis, and sedation, respectively, and ensured prolonged survival up to 22 hours (ref. 29 in R1). No adverse events were identified directly from the specified medication.

Q7. 3. Route of heparin administration – The cited ARDS reference showing potential benefit of heparin used nebulized administration. Please justify why subcutaneous administration was chosen for this study.

**Response**:

Thanks for the comments. The subcutaneous heparin was derived from the reference in adult animal model (ref. 35 in R1). The dosage was based on the most investigational reports for adults and children, following the recommendation from American Pharmacist Association year book (ref. 26 in R1). Due to its half-life in several hours, and being impossible for continuous intravenous injection, a single dose of 100 U/kg BW should be relevant for observation and comparison of its efficacy in parallel or in combination with intratracheal PS and iNO. The measurements of pharmacotherapeutic action are preliminary for understanding its role in the specified neonatal pathological model for clinical implications and construed phenotypes. Due to very low bioavailability, it is not worth delivering heparin alone, or with surfactant as carrier, through aerosol to these tiny pup lungs.

In neonates with central venous access devices (CVADs), a continuous infusion of UFH at 0.5 U/kg/h is recommended to maintain CVAD patency. For umbilical artery catheters (UAC), prophylactic anticoagulation with UFH at a concentration of 0.25-1 U/ml and a total daily dose of 25-200 U/kg/day is recommended. Systemic heparinization (ACT 180-220 sec) is particularly crucial during ECMO [ref. 26, 41 in R1]. As mentioned above in reply to Q3, the use of heparin is relatively safe with regard to the risk of bleeding at therapeutic doses. This point is added in the Discussion of the revised manuscript (lines 552-558, Page 26).

Q8. 4. Validity of the lung injury model – The meconium aspiration group (M group) did not show a higher wet/dry ratio than the control group (C group), raising concerns about whether this model accurately reflects acute lung injury.

**Response:**

Thanks for the comments. In general, the W/D levels reflect postnatal lung fluid clearance efficiency in ventilation, an important physiological variable, being associated, but not directly reflecting, the severity of lung pathology. As mentioned above in reply to Q4, it should be interpreted with LIS and its multiple item scores for severity, and lung weight (LW and LW/BW, which were not available in the protocol), and W/D. Other variables such as PTX, ST_50_, survival time, Cdyn, lung surfactant phospholipids and specific proteins (SP), mRNA expression of alveolar epithelial cells (AT-I and AT-II) and vascular endothelial cells should all be integrated to analyze and interpret the lung injury severity, and their response to the various intervention across the groups. In general, the MAS model should have acute hypoxemic respiratory failure by blood gas during initial ventilation, widespread lung impairment at the time of intervention (2-3 h), and assumed intra- and extrapulmonary shunting and associated pulmonary hypertension. In this study, after 3 hours of mechanical ventilation, the values of Cdyn in M group were significantly lower than that of C group, indicating the validity of the meconium-ALI for subsequent drug treatment. The values of LIS (items and total) do reflect the severity of ALI in those receiving meconium in contrast to the control underwent the same ventilation time with identical ventilator settings (PIP, V_T_).

Q9. 5. Statistical approach – Given the large number of groups and outcome measures, the results are difficult to interpret. Since the primary aim is to determine the effect of adding heparin to standard therapy, the analysis should focus on this aim. Using generalized linear models to assess the independent and interactive effects of each treatment would be more appropriate.
**Response:**

As mentioned above in reply to Q1, the study design was mainly split into two phases: one with ventilation without iNO, and the other with both ventilation and iNO. Thus, the data presented may be interpreted either separately, or in combination, though the statistical analysis was provided for both ways, assuming the random effects across the litters should be basically similar to those within the litters. In separate analyses, four NO groups (MN, MSN, MHN, MSHN) and three non-NO groups (MS, MH, MSH) are statistically compared with their respective control groups (C, M). In R1, we have performed additional statistical analysis and included the test statistics. Additionally, this study conducted Cox regression and correlation analyses for survival time. The results, presented in S9 Table, indicate that treatment (group-wise) served as a protective factor, pneumothorax was a risk factor, and LIS was negatively correlated with survival time. It appears that the four heparin-groups had the lowest hazard ratio after adjustment of confounders. This study compared the ratios of DSPC/TPL values across groups. The results indicate that UFH amplifies the effects of PS, increasing the surfactant pool. The relevant findings are presented in S7 Table in the supporting information files.

Q10. 6. Data presentation – Including figures of selected key parameters (e.g., biochemical markers, qPCR results) would help readers better interpret the findings.

**Response:**

Thanks for this recommendation. In the main text, we have listed key experimental information, such as basic animal data, survival curves, lung compliance, pulmonary histopathological photomicrographs, and phospholipid levels in BALF. Combined with the blood gas analysis, phospholipids and related DSPC/TPL ratios in groups in the total metabolic pool, Cox regression for survival time and partial PCR data in the supplemental materials appendix. This enables evaluation of the efficacy of each medication in this model.

As mentioned above in reply to Q1, we modified the statistical method for intergroup comparisons between any two groups (ANOVA). To better observe the relationship between drug effects, we conducted a pooled-group analysis. The results revealed that UFH significantly improved animal survival rates and reduced TF mRNA expression. Concurrently, PS significantly increased phospholipid content in both animal lung tissue and lavage fluid, while improving blood gas values and alveolar expansion. iNO significantly reduced LIS and decreased inflammatory cytokine mRNA expression while also improving blood gas and alveolar expansion. Based on the comprehensive results of this study, we conclude that UFH demonstrates certain advantages in improving MAS-ALI in neonatal rabbits. Furthermore, its combination with PS and/or iNO had better outcomes compared to single drug use, without causing significant adverse effects.

Other changes:

Title: Newborn is deleted.

S3 Fig, measured mRNA expression values of Ang-1, Ang-2, Tie-1 and Ties-2 from lung tissue are added.
