# Supplementary material for: Efficacy of heparin in respiratory support of near-term rabbits with meconium-induced acute lung injury: Linear regression model analyses
Source: PLoS One. 2026 Mar 24;21(3):e0345718. doi: 10.1371/journal.pone.0345718 (PMC13012467; doi:10.1371/journal.pone.0345718)
Supplement: Attachment — Submitted filename: Response to Reviewers-20260115.docx [file pone.0345718.s010.docx]

Response to Reviewers' comments	20260115

We are very grateful to the reviewers for their further comments regarding the improvement of our manuscript (R1). We have made thorough revision according to the points raised by one of the reviewers, mainly regarding the “generalized linear model”, to characterize the sole and interactive effects of the three medications, and other technical issues in the experiment. We have summarized the response point-by-point below, to the queries (Qx) in order of the decision letter of 202512. In the current version (R2), yellow highlights indicate new or modified content, while red words/fonts denote deleted content referring to the context as marked version.

Review Comments to the Author

Reviewer #2:

Thank you for the opportunity to review the revised version of manuscript entitled “Efficacy of heparin in respiratory support of near-term rabbits with meconium-induced acute lung injury”.

**Q1.** The manuscript has improved following revision; however, the statistical analysis remains inappropriate, and the data presentation is still difficult to follow. As noted below, the data should be analyzed using a generalized linear model to assess the effects of each treatment (UFH, pulmonary surfactant, and NO inhalation) as well as their interactions. Furthermore, this study appears to aim at identifying optimal treatment combinations rather than solely evaluating the efficacy of UFH. Please consider revising the entire manuscript, including the title, to clearly reflect this objective.
**Response:** Thanks for the comments. We analyzed part of the data using generalized linear models, as detailed in below response to question 4 (**Q4**).

In the current revision (R2), we have conducted a generalized linear regression analysis with survival status as the primary outcome (Y) in the model as a dependent variable [f (Y)]. By incorporating all main effects of PS, UFH, and NO as well as all possible two-way and three-way interaction terms (i.e., a full factorial model), we stepwise characterized their respective effects. The analysis used other variables derived at the end of survival, with variable ventilation length, LIS_total_, Vv, CV (Vv), DSPC_BALF_, pH, PCO_2_, LAC (in heart blood), W/D, Cdyn_mean_ (derived from the last 7 hours during the 10-h total ventilation length) instead of survival length. Our major findings suggest that:

1) Both PS and NO exerted significant independent main effects on LIS_total_, each with an overt reduction of LIS_total_. However, significantly negative interactions were observed between NO×PS and NO×UFH, indicating that their combined effects were less than the sum of their individual effects. NO alone exerted the strongest reduction in LIS_total_. Based on comparisons of marginal means, the concurrent use of PS and NO (MSN group) or UFH and NO (MHN group) resulted in a lower LIS_total_ than the use of UFH and PS alone (S10.1- S10.3 Table).

2) Main effects of PS, UFH, and NO all showed significant positive associations with Vv, while a significant negative interaction was observed between PS and UFH, indicating that their combined use resulted in a smaller increase in Vv than the sum of their individual effects (S10.4- S10.6 Table).

3) The main effects of PS, UFH, and NO were each significantly associated with a decrease in CV (Vv). This indicates that the effects of these three factors on CV(Vv) are primarily independent and additive (S10.7- S10.8 Table).

4) While each factor was a positive regulator of pH as a single outcome item, pairwise combination comparisons showed mutual antagonism. The concomitant administration of all three, however, revealed a synergistic/compensatory mechanism that partly overcame this interactive antagonistic mechanism, thereby resulting in significantly elevated pH values (S10.9- S10.11 Table).

5) PS and NO were independent factors that lowered PCO₂ (S10.12-S10.13 Table). PS was an independent factor that lowered LAC (S10.14-S10.15 Table). PS was an independent factor for higher DSPC_BALF_ (S10.16-S10.17 Table)_._

6) UFH was a significant protective factor, associated with a significantly reduced risk of mortality (S10.18-S10.19 Table).

7) Only NO exerted an independent main effect that significantly lowered W/D levels (S10.20-S10.21 Table).

8) Both PS and UFH, as well as NO, can independently improve Cdyn_mean_. However, a significantly negative interaction existed between PS and UFH when used in combination (S10.22-S10.23 Table).

We summarized above finding in the section of Methods/ statistical analysis (lines 296 to 303 on page 14 of R2), Results/Regression models (lines 489 to 508 on page 24 of R2), and Discussion/implications (lines 528 to 549 on page 25 of R2) of survival analysis using the generalized linear regression models, and amended the Cox HR regression for survivals (lines 456 to 459 on page 21 of R2). To facilitate reviewers' evaluation, a separate file entitled "S10.1-S10.24 Tables," which details each generalized linear regression model, is attached as supplemental material with this revision (R2). As a result, we have modified the article title of R2 to be more informative, highlighting the “generalized linear regression model” in the estimation of efficacy.

There is a paucity of other medications in addition to the PS and/or iNO regimens in the literature. Likewise, UFH therapy for MAS remains underexplored in existing literature, though it was reported in pediatric and adult respiratory and critical care, including preclinical experimental pharmacology and pathophysiology of ALI (refs. 41, 47, 49 in R2). Therefore, this study focused on evaluating the pharmacodynamic response of UFH in MAS, with particular attention to potentially synergistic interactions with PS and/or NO. In the Results and Discussion sections of R1, we only addressed the synergistic effects from the phospholipid components in BALF and LH compartments. We did find the impact of UFH on the phospholipid metabolic pools (lines 433 to 438 on page 20 and lines 603 to 606 on page 28 of R2). Specifically, our findings demonstrate that UFH and PS exhibit a synergistic effect, and similarly, UFH and iNO also act synergetically.

We modified and re-analyzed the Cox hazard ratio (HR) survival regression models, while BW, Cdyn_mean_, and LIS_total_ were valid parameters to be included; therefore, this table should be retained in R2, supplemental S9 Table, for comprehension.

**Q2.** Intravenous lidocaine alone cannot provide general anesthesia. It appears that no other general anesthetic agents were administered to the neonatal rabbits. Please justify why lidocaine-only anesthesia was considered appropriate.

**Response:** Thanks for the comments.

The does received sedatives (diazepam) and anesthetics (urethane, repeated dosage) should have impacted the fetuses before delivery (ref. Meissner J, Preil P. Distribution of 14C-Diazepam (Valium) in pregnant and lactating rabbits. Nucl Med (Stuttg). 1975 Aug 31;14(3):272-84; Kanto JH. Use of benzodiazepines during pregnancy, labour and lactation, with particular reference to pharmacokinetic considerations. Drugs. 1982 May;23(5):354-80; Field KJ, Lang CM. Hazards of urethane (ethyl carbamate): a review of the literature. Lab Anim. 1988 Jul;22(3):255-62).

In the 1980s-1990s, a combination of acepromazine and ketamine or sodium pentobarbital and pancuronium bromide were used for general anesthesia of neonatal rabbits (ref. 7, 8, 20, 32 in R2). These regimens were employed in short-term (30 minutes to 5 hours) ventilation studies in newborn rabbits at 27-30 days of gestation (full-term gestation: 31 days). However, due to the immature metabolism/catabolism in immature rabbits, these anesthetic protocols frequently caused respiratory depression, making them unsuitable for long-term survival observations. In the latter 1980s, investigators tended to apply a mild anaesthesia without additional i.p. anaesthesia, but pacuronium in some, in the newborn rabbits subjected to the experiment with short-time (60 min) mechanical ventilation (Fiscone JM, Jacobs HC, et al. Pediatr Res 1987; 22(6): 730-735; Fiascone JM et al. Exp Lung Res 1989; 16: 311-321; Mercury MR et al. J Appl Physiol 1989; 66(5): 2039-2044).

Based on our previous experience with ventilation studies in newborn rabbits (gestational age 26-30 days), does received intramuscular injections of diazepam and urethane for sedation and anesthesia, followed by intravenous supplementation of urethane to maintain the doe anesthesia till completion of delivery. Like other anesthetics for general anesthesia, diazepam and urethane can cross the placental barrier within a short period, resulting in newborn pups being born in a semi-anesthetized state. Immediately after birth, initial anesthesia was achieved via intraperitoneal injection of diluted lidocaine solution, supplemented by subcutaneous infiltration of diluted lidocaine for local anesthesia around the front neck during tracheal intubation to the pups when necessary. Throughout the ventilation period, a mixed solution containing 2% lidocaine, 5% NaHCO₃, and 10% glucose (vol/vol/vol 1:3:6 or 2:3:5) was administered every 1-2 hours to each pup. This combination provided triple pharmaceutical actions: glucose for energy supply, NaHCO₃ for correction of metabolic acidosis, and lidocaine for anesthesia.

The protocol for mechanically ventilated newborn rabbits was construed to simulate clinically critical care with minimum stimulation and avoid drug adverse effects. Thus, it exerts mild anesthetic effects while the spontaneous respiratory effort shown as chest and abdominal movement during mechanical ventilation was maximally preserved, for prolonged survival (ref. 27-31 in R2). During mechanical ventilation, for those with good response, spontaneous breath movement, with no distress-induced body movements were observed, along with stable heart rates (150-250 beats/min) throughout (unless early death occurred). Direct adverse events attributable to these pharmacological agents were closely observed (pneumothorax, ECG tracings) (ref. 27-31 in R2). For those with poor response from the initial 15-60 min of ventilation, they tended to be pale or cyanotic in the head, body and limbs, lower heart rate alteration (50-100/min), with ECG S-T fragment alteration denoting cardiac ischemia and/or atrioventricular block. They may survive shortly with transient improvement in oxygenation. These were found mainly in those who died within the initial 1-2 hours.

From one of the coauthors (B.S)’s experience in the 80s and 90’s when both pentobarbital and pancuronium were used, the preterm animals (GA27d) may survive for only 30-60 min, which was the time limit in most of the studies then using preterm rabbits at GA 27-27.5 days, with or without ECG monitoring. Their ventilation was often with very high tidal volume (10-12 ml/kg) resulting in a very fast heart rate (250-300/min, being tachycardia), which was not physiologically acceptable from current knowledge of lung protective ventilation strategy in neonatal critical care. Likewise, in the studies with pups of GA in 29/29.5/30 days, pentobarbital and pancuronium may also cause adverse effects in cardiopulmonary function during mechanical ventilation. Thus, the analgesic use with lidocaine in our experiments was based empirically on the maternal general anesthesia to fetuses, tracheostomy at birth as resuscitation to avoid hypoxic fatality, and comfort during prolonged ventilation, which seemed amenable within the modified study protocol. This technical limitation is now added in the discussion-limitation, lines 636-640 on page 29 of the R2 marked version. A short phrase is added as “subcutaneous infiltration of the anterior neck with the same lidocaine, when needed” in R2-marked version, Materials and methods, Animal management and MAS model (lines 148-151 on page 7).

**Q3.** Why were the animals ventilated with 2–3 cmH₂O PEEP? This level seems relatively low and may predispose to lung collapse. Please explain the rationale for selecting these PEEP settings.

**Response:** Thanks for the comments.

Since the 1970s and 1980s, investigators have initiated mechanical ventilation in preterm and near-term rabbits at GA 27-30d. They observed that epithelial cell damage, associated with alveolar structure immaturity, surfactant deficiency, and uneven alveolar expansion collectively contributed to acute lung injury, which constituted the underlying pulmonary pathology of respiratory distress syndrome (RDS) in the preterm rabbits (Nilsson R, Grossmann G, Robertson B. Pediat Res 1978; 12:249-255; Nilsson R. Acta Anaesthesiol Scand. 1982 Apr;26(2):89-103). At that time, the equipment was already capable of providing mechanical ventilation to multiple animals in parallel for testing surfactant in the improvement of lung mechanics, i.e., dynamic compliance of respiratory system (Cdyn, or Crs). In those studies, PEEP was not used to avoid masking the surfactant effects. Moreover, the pressure entering each animal lungs could not be individually adjusted (Lachmann B. Pediatr Res 1981; 15:833-838; 1982; 16:921-927). In the meantime, others reported individually adjusted PIP-PEEP based on “one ventilator-to-one pup” settings (Tooley W, Clements JA, et al. Am Rev Respir Dis 1987; 136:651-656), which set ventilator PEEP only 1 cmH_2_O with very fast frequency (48 beat/min) to achieve (supposedly with intrinsic) PEEP at 4 cmH_2_O, and achieved very high tidal volume, up to 10-15 ml/kg within 60 min.

Lately, one of the co-authors (BS) working at Karolinska experimental perinatal pathology laboratory (Head, Dr. Bengt Robertson), modified the equipment to enable pressure regulation for each individual animal, achieving a ventilation mode with target tidal volume (Vt) with PIP-PEEP. This emphasized the importance of animal models that conform to respiratory physiology for comparing the effects of different surfactant preparations. (**Sun B**, Kobayashi T, Cursted T, Grossmann G, Robertson B. Eur Respir J. 1991;4:364-70).

Subsequently, it revealed that appropriate PEEP (2-3 cmH₂O) promotes alveolar recruitment in GA27d preterm rabbits without increasing the incidence of pneumothorax (was up to 50%), thereby facilitating the establishment of mechanical ventilation models with PIP-PEEP restricted tidal volume below 9 ml/kg (Rider ED, Jobe AH, Ikegami M, **Sun B**. J Appl Physiol 1992; 73(5): 2089-2896). This was in contrast to their previous studies with no PEEP but very large tidal volume of 10-15 ml/kg by individually adjusted PIP in testing efficacy of perinatal medications including surfactant preparations in preterm rabbits (Ikegami M, Jobe AH, et al. J Clin Invest 1987; 79:1371-1378; Am Rev Respir Dis 1987; 136:892-898).

The multi-channel mechanical ventilation model underwent further refinement, enabling a series of studies on the pathogenesis and intervention efficacy of diseases associated with preterm-to-near-term rabbits with bacterial pneumonia, or meconium aspiration (**Sun B**, Robertson B et al. Biol Neonat 1993; 63:96-104; Herting E, **Sun B**, Robertson B, et al. Pediatr Res 1994; 36:784-791; ref. 30 in R2).

Previously reported data from our team indicate that PEEP modifies the response of preterm rabbits to surfactant during ventilation (ref. 30 in R2). In recent years, our research group has successfully established a multi-channel parallel mechanical ventilation model in newborn rabbits at gestational days 26–30, simulating human lung development in third trimester: across the late canalicular, early and late saccular, and early alveolar stage before term birth, and alveolar stage at early postnatal life (Zhuang G et al, Exp Biol Med 2026 accepted). These were achieved by optimizing Vt at 4-6 mL/kg and PEEP at 2–3 cmH₂O (ref. 27, 28, 30 in R2). Given the parallel ventilation design of our model (supporting up to 12 animals simultaneously), the flow partitioning effect may lead to progressive pressure decay during mechanical ventilation. Additionally, pressure adjustments carry inherent risks of air leakage due to interaction among the gas flow outlets for maintenance of PIP-PEEP level in gradient in individual units, within the plethysmograph-ventilator circuit that was provided with a very high main gas flow from the ventilator. While we may not achieve PEEP to 4-5 cmH₂O, the system can maintain relatively stable PEEP levels at 2-3 cmH₂O across all animals on parallel ventilation. Integrated with a protocol of NICU-mimicking, comprehensive management, this model effectively recapitulates critical developmental transitions in the lung maturation. This technical limitation is now added in the discussion-limitation, on page 30 lines 641-647 of the R2 marked version.

**Q4**. Although the information on group allocation has improved, the statistical analysis remains inadequate. This study evaluates the efficacy of three interventions (UFH, pulmonary surfactant, and NO inhalation) and their interactions in an MAS model. A generalized linear model would be more appropriate. Please consider reanalyzing all data using generalized linear analysis. In addition, the approach to variable selection in the multivariate Cox regression model appears inappropriate. The analysis should assess the effect of each treatment and their interactions.

**Response:**

We appreciate very much for this proposal. We performed a generalized linear regression analysis. The detailed data are provided in a separate attachment as supplemental materials (S10.1-S10.24 Table). There are 8 groups modeled with meconium aspiration and assigned "1" for drug use and "0" for no use. Groups were coded based on different drug combinations; for example, MSHN was coded as (1,1,1). A generalized linear model incorporating the three factors (PS, UFH, NO) was established, with subsequent pairwise comparisons corrected via the Bonferroni method.

The model analysis revealed that the independent main effects of PS and NO on LIS_total_ were significant, with both reducing the LIS_total_. However, significantly negative interactions were observed for both NO×PS and NO×UFH, indicating that their combined effects were sub-additive (i.e., less than the sum of their individual effects). NO alone produced the most potent reduction in the LIS_total_. Comparisons of marginal means showed that the LIS_total_ in the MSN group was lower than that in the MS group (mean difference = -1.87, *p* = 0.025). Similarly, the LIS_total_ in the MHN group was lower than that in the MH group (mean difference = -1.87, *p* = 0.025). These findings suggest that despite the antagonism between the agents, an additive or saturation effect existed, where the two-drug combination seemed to approach a ceiling of efficacy.

The main effects of PS, UFH, and NO all had a significant positive impact on the higher Vv and the lowered CV(Vv). Regarding interactions, PS×UFH showed a significantly negative interaction effect on Vv (B = -0.096, *p* = 0.004), suggesting that their combined effect on Vv was a smaller increase than the sum of their individual effects.

The regulatory mechanisms of PS, UFH, and NO on pH were highly complex, involving a significant three-way interaction. Each factor alone elevated pH, but pairwise combinations exhibited antagonistic effects. However, when all three coexisted, a synergistic or compensatory mechanism emerged, partially reversing the antagonistic effects observed in dual combinations, and which resulted in a final pH higher than what may be predicted by a simple antagonistic model. For instance, when UFH = 1, the pH in the [PS = 1, NO = 1] group was significantly higher than that in the [PS = 1, NO = 0] group (*p* = 0.043), indicating that adding NO in the presence of PS and UFH elevated pH, a manifestation of the three-way interaction.

PS and NO were independent factors in reducing PCO₂, and PS was an independent factor in reducing LAC. PS had a significantly independent main effect on DSPC_BALF_ levels, with its presence associated with significantly elevated DSPC_BALF_ levels (B = 0.823, 95% CI: 0.420 to 1.227).

Generalized linear regression analysis (dependent variable: 0 = survival, 1 = death) showed that only UFH had a significant independent main effect on mortality risk (likelihood ratio Chi-square = 6.352, *p* = 0.012). The use of UFH (UFH = 1) was a significant protective factor, associated with a significantly reduced risk of death, with an odds ratio (OR) of 0.105 (95% CI: 0.005 to 0.687).

Only NO had a significantly independent main effect on W/D levels. The presence of NO (NO = 1) was associated with a significant reduction in W/D levels (B = -0.643, 95% CI: -1.262 to -0.024).

The model analysis revealed that PS, UFH, and NO each independently improved Cdyn_mean_. However, a significantly negative interactive effect was observed between PS and UFH, indicating that their combined benefit was lower than expected. When PS or UFH was held constant, the addition of NO did not confer a significant additional benefit to the improvement in Cdyn_mean_. This finding reinforces the notion that the effect of NO may be “masked” or “saturated” by the presence of either PS or UFH.

The overall test for the three-way interaction among PS, UFH, and NO did not reach statistical significance for LIS_total_, Vv, CV(Vv), PCO₂, LAC, DSPC_BALF_, survival outcome, W/D, or Cdyn_mean_. Whether higher-order interactions exist among the three interventions requires further validation in studies with larger sample sizes.

We included variables associated with survival time and significant in univariate Cox analysis into the model, controlled for body weight as a confounder, and ultimately established a Cox regression. In the final table, Cdyn_mean_ was included (r = 0.339, *p* <0.001), while Vv and DSPC_LH+BALF_ were removed due to lack of correlation with survival time.

By consulting statistician, we looked at reference books for MANOVA and ANCOVA (in Norman GR & Streiner DL: Biostatistics. The Bear Essentials, 4^th^ Ed., 2014, People’s Medical Publishing House, Shelton, Connecticus, USA), and for multiple variable regression (Chapter 12, analysing multiple variables per subject, multiple regression and analysis of variance (including two-way analysis of variance), and logistic regression, in Peacock JL and Peacock PJ: Oxord Handbook of Medical Statistics, 1^st^ Ed. 2011, Oxford University Press, New York, USA).

We understand the reviewer’s proposal to use generalized linear models to analyze the effects across the intervention groups. Therefore, the corresponding revisions have been made and can be found on page 14, lines 296-303; page 24, lines 489-508; and page 25, lines 528-549 of R2. Given the limited sample size, fragmented sampling methodology, and discontinuous data in the present study, the interpretation of these analyses should be approached with considerable caution. As our subjects were at GA 30 days with variable birth weight (BW) and gender (sex) that may be confounders or covariates (covariables) in reference to the treatment groups as independent variables, we did three rounds for multi-variable logistic regression models. In the tables, represent groups with combined treatment as non-PS vs. all-PS, non-UFH vs. all-UFH, and non-iNO vs. all-iNO, respectively, the results from logistic regression models do not suggest that BW would affect the outcome whereas the Cdyn_mean_ was a significant one that seems to exert protective effects. Likewise, by comparing the M group, each of the seven treatment groups (MS, MH, MSH, MN, MSN, MHN and MSHN) did not have any co-variables that should account for the outcome. We noticed that two-way ANOVA requires repeated measure within the time course whereas our datasets did not have strictly time course defined subsets to compare, neither did it have sufficient sample size to conduct. This test was not performed while we prepare this work to respond. The gender/sex was a dichotomous variable and is not suitable for ANCOVA use. The BW should be one of the independent covariable to affect the ourcome (survival). We checked its correlation with the survival time of each group, and found there was no correlation of BW (r = -0.095, *p* = 0.200), but a mild correlation of Cdyn_mean_ (r = 0.339, *p* < 0.001), with the survival length. The determination of coefficient of the latter (r^2^ = 0.115) also suggests it not to be an valid covariable for ANCOVA analysis.

**Q5.** Please include PaO₂ data from the blood gas analysis.
**Response:** Thanks for the comments. Per our protocol, left ventricular blood was collected via open-chest puncture immediately after weaning from mechanical ventilation for blood gas analysis. While left ventricular blood theoretically represents proximal aortic blood and should reflect systemic arterial blood gas values, the measured partial pressure of oxygen (PaO₂) was consistently lower than expected (observed range: 9-14 mmHg in this study) and thus excluded from final analysis. We assume this discrepancy were associated with transient hypoventilation post-weaning and alveolar collapse from loss of thoracic negative pressure after thoracotomy, leading to reduced alveolar oxygenation through effective gas exchange. Technically, due to the small heart size - inadvertent deep needle penetration or misplacement could be mixed with left atrial or right ventricular blood as mixed blood sample. In contrast, pH, partial pressure of carbon dioxide (PCO₂), and lactate levels remained stable with minimal short-term fluctuations, attributable to systemic metabolic homeostasis and efficient buffering capacity. Like the previous publications, we do not consider to include PO_2_ (PaO_2_) as the valid result in the S2 Table.

**Q6.** Were all animals administered meconium from the same batch? Please clarify this point.

**Response:** Thanks for the comments. We collected adequate fresh meconium (dark green stools) from healthy full-term neonates within 24 hours after birth. The meconium was mixed with sterile water and evenly spread on glass Petri dishes. Samples were sealed with plastic film and frozen overnight at -20°C. The following day, the meconium-coated dishes were lyophilized using a vacuum freeze-dryer. After lyophilization, the meconium was ground into a homogeneous powder and stored at -20°C. For this study, all experiments used the same batch of meconium, with fresh suspensions prepared daily. For example, on the day of experimentation, 100 mg of meconium powder was reconstituted in 1 mL of normal saline (0.9% NaCl) to achieve a standardized concentration of 100 mg dry weight/mL.
